# Combined Analysis of the Time-Resolved Transcriptome and Proteome of Plant Pathogen *Xanthomonas oryzae* pv. *oryzae*

**DOI:** 10.3389/fmicb.2021.664857

**Published:** 2021-06-02

**Authors:** Seunghwan Kim, Wooyoung Eric Jang, Jihwan Park, Min-Sik Kim, Jeong-Gu Kim, Lin-Woo Kang

**Affiliations:** ^1^Agricultural Microbiology Division, National Institute of Agricultural Sciences (NAS), Rural Development Administration (RDA), Jeonju, South Korea; ^2^Department of Chemistry, Kyung Hee University, Gyeonggi-do, South Korea; ^3^Department of New Biology, DGIST, Daegu, South Korea; ^4^Genomics Division, National Institute of Agricultural Sciences (NAS), Rural Development Administration (RDA), Jeonju, South Korea; ^5^Department of Biological Sciences, Konkuk University, Seoul, South Korea

**Keywords:** *Xoo*-rice interactions, proteome and transcriptome, time-resolved gene expression, *Xanthomonas oryzae* pv. *oryzae*, pathogenicity, translational regulation

## Abstract

*Xanthomonas oryzae* pv. *oryzae* (*Xoo*) is a plant pathogen responsible for causing bacterial blight in rice. The immediate alterations in *Xoo* upon initial contact with rice are essential for pathogenesis. We studied time-resolved genome-wide gene expression in pathogenicity-activated *Xoo* cells at the transcriptome and proteome levels. The early response genes of *Xoo* include genes related to cell motility, inorganic ion transport, and effectors. The alteration of gene expression is initiated as early as few minutes after the initial interaction and changes with time. The time-resolved comparison of the transcriptome and proteome shows the differences between transcriptional and translational expression peaks in many genes, although the overall expression pattern of mRNAs and proteins is conserved. The discrepancy suggests an important role of translational regulation in *Xoo* at the early stages of pathogenesis. The gene expression analysis using time-resolved transcriptome and proteome provides unprecedented valuable information regarding *Xoo* pathogenesis.

## Introduction

Rice (*Oryza sativa* L.) is the most widely consumed staple food, sustaining two-thirds of the world’s population (Jackson, 2016). *Xanthomonas oryzae* pv. *oryzae* (*Xoo*) is a causal agent of bacterial blight of rice, which causes severe yield losses of up to 50% in several rice-growing countries (Oliva et al., 2019). The demand for rice is expected to increase by at least 25% by 2030, owing to the rapidly growing world population, environmental stress arising in response to climate change, and pathogen pressure (Li et al., 2014). The Green Revolution has resulted in a shift in rice cultivation, from varied traditional landraces to limited high-yielding varieties, via artificial selection. This has caused the co-evolution of crop pathogens including *Xoo* with the selected host races in the modern agricultural ecosystem (Quibod et al., 2020).

The pathogen-host system of *Xoo* and rice serves as an ideal agricultural model to study crop diseases in a field setting at the molecular level; this is facilitated by the early elucidation of the whole genome structure of both *Xoo* and rice (Lee et al., 2005; Jackson, 2016). *Xoo* typically invades rice leaves through the wounds or hydathodes and replicates in the xylem vessels to cause disease (Mew et al., 1993). Rice contains a two-tiered innate immune system, consisting of pathogen-associated molecular pattern- and effector-triggered immunity, which protects against *Xoo* and initiates the hypersensitivity response at the infection site (Jones and Dangl, 2006). In *Xoo*-rice interactions, *Xoo* injects effectors into rice cells to modulate the cellular activities of the host to promote pathogenesis (Tsuge et al., 2014). The early interactions between *Xoo* and rice at the infection site determine the fate of infection, i.e., occurrence of disease or initiation of the immune response. The environmental conditions prevalent at the site of infection are varied and complex, and our understanding of alterations in the *Xoo* cells in response to the initial interactions with rice is limited.

Transcription and translation are tightly coupled in bacteria and can occur simultaneously in the cytosol (Hershey et al., 2019). Although proteins are the final functional products of genes, the quantity of specific mRNA molecules often represents the expression level of a gene at a given time point with the well-established RNA sequencing (RNA-seq) technology. In comparison, high resolution mass spectrometry-based quantitative proteomics is a more recent analytical technique, and still has a lower coverage of protein products, requires greater sample quantity, and is more expensive (Schubert et al., 2017).

We had previously developed an *in vitro* pathogenicity assay to recapitulate *Xoo*-rice interactions at the site of infection by treating *Xoo* cells with the rice leaf extracts (RLX), and assessed the time-resolved changes in the transcriptome (Kim et al., 2011, 2013, 2016). The *in vitro* pathogenicity assay provides high signal to noise data with *Xoo* cells synchronized with respect to the timing of pathogenicity activation. Transcriptome data from RNA-Seq experiments revealed that most virulence genes of *Xoo* were upregulated within an hour of the initial interaction with RLX, and these upregulated genes were related to bacterial motility, inorganic ion transport, hypersensitive response and pathogenicity (*hrp*), bacterial toxins and effectors of avirulence (*avr*), plant cell wall degradation, and extracellular polysaccharide synthesis and secretion (Kim et al., 2016).

Here, we expand the study of gene expression in pathogenicity-activated (P-activated) *Xoo* from the transcriptome to the proteome. The time-dependent expression of specific mRNAs and proteins represents the kinetics of transcriptional and translational expression of certain genes and allow the visualization of the immediate alterations in gene expression in P-activated *Xoo*.

## Results

### Time-Resolved Proteome Data

**SCHEME 1:**
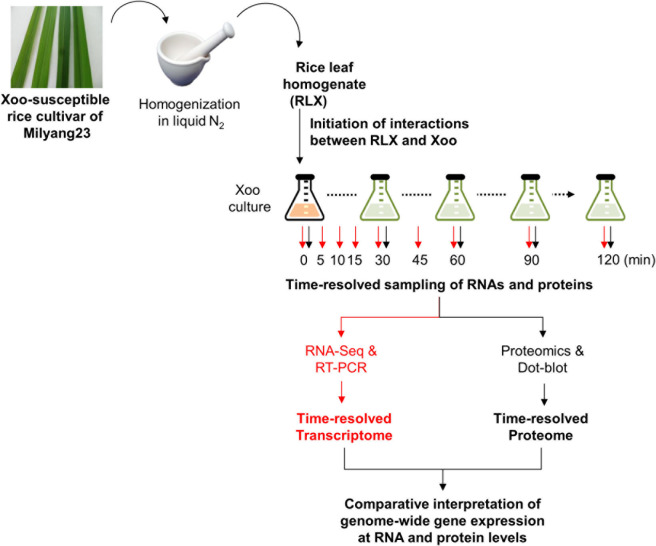
Schematic representation of the *in vitro* assay system and combined analysis of the time-resolved transcriptome and proteome using RNA-Seq and LC-MS/MS.

We coupled LC-MS/MS technology with an *in vitro* assay system to obtain the time-resolved proteome data for P-activated *Xoo* cells (Scheme 1). The *in vitro* assay system recapitulated the initial interaction between *Xoo* cells and damaged rice leaf tissues at the site of infection. Fresh RLX were prepared by grinding the leaves of a *Xoo*-susceptible rice cultivar (Milyang 23) in liquid nitrogen and added to a *Xoo* cell culture in the mid-exponential phase. Samples for proteome analysis were collected from RLX-treated (P-activated) and untreated (control) *Xoo* cells at 0, 30, 60, 90, and 120 min after RLX treatment (Supplementary Table 1).

Analysis on UniProt revealed that the total 4,956 predicted open reading frames in the *Xoo* genome (KACC10331) corresponded to 4,382 proteins in the proteome, of which 2,589 proteins (59%) were identified for at least one time point and 2,296 proteins (52%) were detected in both replicates (Supplementary Figure 1A). Median sequence coverages for total identified proteins were 24% and 23% for each of the independent duplicate experiments (Supplementary Figure 1B). A total of 20,963 and 19,684 non-redundant peptides were identified in both replicates, with 47,025 and 41,428 peptide-spectrum matches, respectively (Supplementary Figure 1C).

Protein abundance values obtained after quantile-normalization (Supplementary Figure 2A) were used for pairwise comparisons; the Pearson’s correlation coefficients corresponding to the abundance values showed close correlations (0.98–0.99), indicating comparable cellular concentration of most proteins (Supplementary Figure 2B). The smallest correlations were observed for the RLX-treated samples at 30 min, indicating greater changes in the proteome during the initial 30 min; this was consistent with the transcriptome data. We further performed a principal component analysis to determine the relationships between the assessed samples. Supplementary Figure 2C shows that the P-activated sample at 0 min clustered closely with all control samples, whereas the P-activated samples at other time points were more spread out. The proteome of the P-activated *Xoo* cells at 30 min was considerably different from that of P-activated *Xoo* at other time points; this was consistent with the results of the pairwise multi-scatter plot (Supplementary Figure 2B).

### Up and Down Regulated Proteins

**FIGURE 1:**
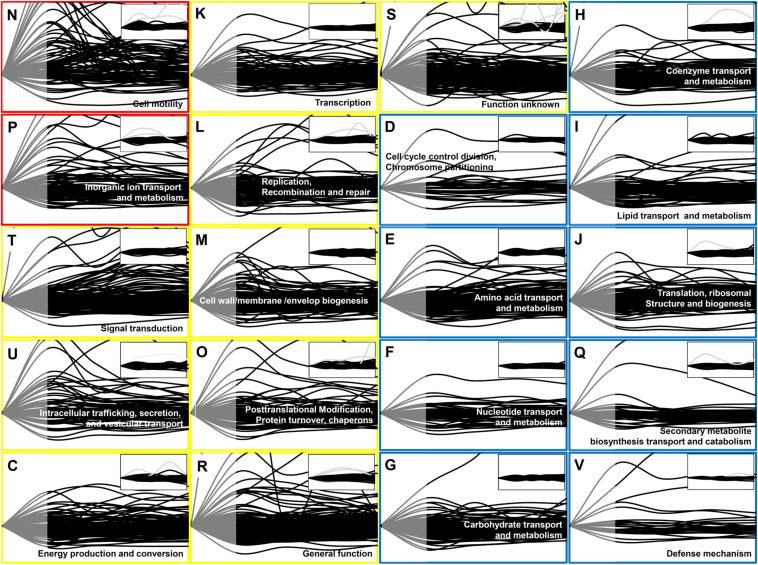
Time-resolved protein expression patterns associated with the COG categories. Two functional categories of genes (COG) associated with the greatest changes in the protein level are indicated by red boxes, nine functional COGs associated with moderate changes are indicated by blue boxes. One-letter abbreviation and name for each functional COG are indicated in each box. The inset for each functional COG indicates the control (untreated cells). The *Y*-axis represents the fold change in the protein expression level in comparison with that at 0 min, and the maximum value on the *Y*-axis was set as 3. The *X*-axis indicates the time from 0 to 120 min. Each line indicates the protein expression level of a gene. The protein expression level is represented as gray (from 0 to 30 min) and black (from 30 to 120 min) lines. Note that in contrast to the transcript data, the proteome data was not available between 0 and 30 min (gray part).

Quantitative proteome data obtained from P-activated and control *Xoo* cells at every 30 min allowed the visualization of the three-dimensional protein expression data in terms of the genes, time intervals, and expression levels (Figure 1 and Supplementary Table 2). The expressed protein level of each gene from P-activated *Xoo* cells are divided by that of the same gene from control at each time point to calculate the fold change of time-dependent protein expression level of the specific gene (Supplementary Table 3). For all the open reading frames, approximately 93 (2.0%), 213 (4.5%), and 468 (9.9%) proteins were upregulated by more than 200% (two-fold), 50%, and 20% at 30 min, respectively, and approximately 7 (0.1%), 93 (2.0%), and 561 (11.9%) proteins were downregulated to less than 25% (two-fold), 50%, and 80% at 30 min, respectively.

In case of the duration of expressed proteins in P-activated *Xoo*, only 8 (0.2%), 32 (0.7%), and 75 (1.6%) proteins were upregulated for entire 120 min by more than 200% (two-fold), 50%, and 20%, respectively, and 0 (0%), 1 (0.02%), and 87 (1.8%) proteins were downregulated for the same 120 min to less than 25% (two-fold), 50%, and 80%, respectively (Supplementary Table 4), indicating that most proteins were temporarily upregulated or downregulated in the *in vitro* assay.

The proteins at 30 min presented the highest change at the expression level within 120 min. More than 90 proteins (1.9%) were upregulated by more than two-fold at 30 min in each dataset, whereas 27 proteins were upregulated in both datasets from the duplicate experiments (Supplementary Table 5). Almost half of the 27 upregulated proteins were related to cell motility and ion uptake, seven were related to chemotaxis and motility, and five were related to transporters and pumps. At the same 30 min, more than 90 proteins were downregulated by more than 50%, 17 proteins were downregulated in both datasets (Supplementary Table 6), including transcription-related proteins—such as sigma-54 modulation protein and MetE/MetH family transcriptional regulator—and cell division- and cell cycle-related proteins.

### Clusters of Orthologous Groups of Proteins (COGs)

To study the global gene expression pattern based on gene function, we superimposed time-dependent protein expression levels as per the functional categories in the Clusters of Orthologous Groups of proteins (COGs) database (Figure 1), which were grouped into three classes (red, yellow, and blue) depending on the observed pattern. To facilitate the comparison of time-dependent protein expression levels, the expression level at 0 min was set as the reference level (=1) for each gene, and the fold change in the protein expression level was calculated at each time point, as in case of the RNA-Seq data analysis (Supplementary Table 2; Kim et al., 2016).

**FIGURE 2:**
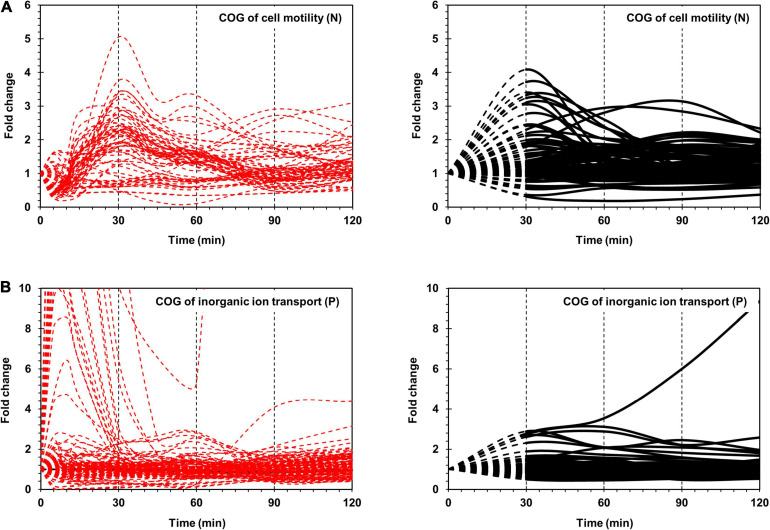
Time-resolved mRNA and protein levels of genes associated with cell motility and inorganic ion transport. **(A)** Time-resolved expression of cell motility-related genes. The mRNA expression levels from RNA-Seq are indicated by dashed red lines. The protein expression levels from LC-MS/MS are indicated by black dashed (from 0 to 30 min) and black solid (from 30 to 120 min) lines. **(B)** Time-resolved expression of inorganic ion transport-related genes. The mRNA expression levels from RNA-Seq and protein expression levels from LC-MS/MS are indicated as in **(A)**. The *Y*-axis represents the fold change.

The most prominent changes in protein expression in *Xoo* cells were observed for proteins associated with cell motility (N) and inorganic ion transport and metabolism (P), which were placed in the red class (Figure 2). In category N, two major expression peaks, indicating more than two-fold upregulation, were detected at 30 and 90 min. In category P, upregulated proteins peaked at around 30 min. Proteins grouped in the yellow class exhibited moderate changes in their expression level, and were divided into nine functional categories, including signal transduction (T), intracellular trafficking, secretion, and vesicular transport (U), energy production and conversion (C), transcription (K), replication (L), cell wall/membrane/envelop biogenesis (M), and protein turnover (O). The blue class included proteins associated with other nine categories, the expression levels of which exhibited little change when compared with those at 0 min, albeit except for a few proteins.

### Time-Resolved Proteome and Transcriptome Analysis

We integrated two different molecular levels of mRNAs and proteins to investigate the time-resolved systematic analysis of both transcriptome and proteome in P-activated *Xoo* cells. Both transcriptome and proteome data were obtained for up to 120 min after RLX treatment (Scheme 1). The experimental procedure used to prepare protein samples for LS-MS/MS required at least 30 min, whereas that for mRNA samples required at least 5 min; the shortest interval of time for the proteome data was set to 30 min, whereas that for transcriptome data was set to 5–30 min. Among the protein-coding genes observed in both proteome and transcriptome, we selected 1,868 genes which were detected more than three times in both omics layers. The integrative expression matrix was generated by merging two separate expression *z*-score matrices of mRNAs and proteins.

**FIGURE 3:**
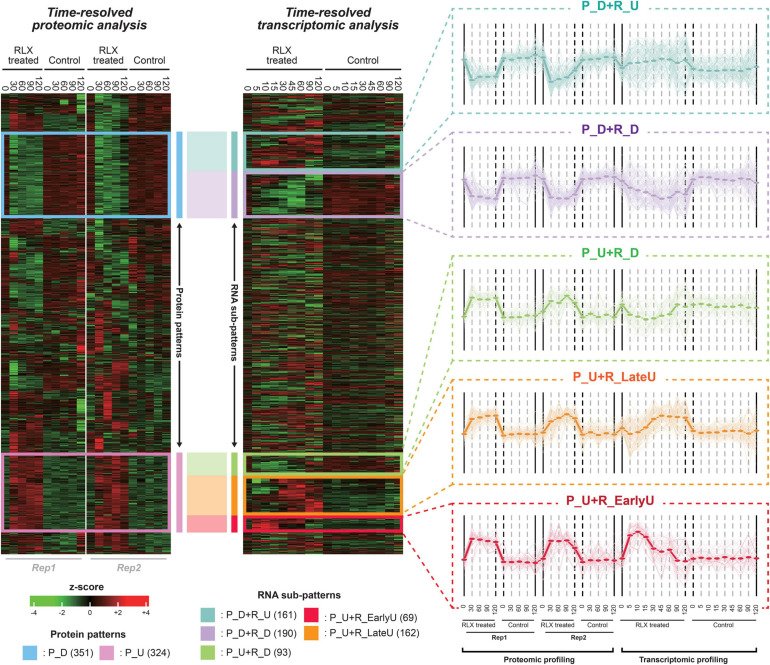
Time-supervised hierarchical clustering of pathogenicity-activated proteome and transcriptome datasets. The left and right heatmap shows protein and mRNA expression profiling of P-activated and control *Xoo* cells, respectively. The blue and pink box in left heatmap represent down(P_D)- and up(P_U)-regulated proteins in P-activated *Xoo* cells. The turquoise, lavender, lightgreen, yellow and red in right heatmap represent sub-patterns (P_D + P_U, P_D + R_D, P_U + R_D, P_U + R_EarlyU and P_U + R_LateU) in time-resolved mRNA levels. Low to high expression is indicated by a change in color from green to red. For each sub-pattern, the expression patterns from integrative expression matrix are shown in the right side. Expression profiling of each gene in sub-pattern is presented by light thin lines. The average values of expression profiling of sub-pattern are presented by thick lines.

After performing the hierarchical clustering analysis to the integrative expression matrix, we found out two major proteomic patterns of upregulated and downregulated protein levels (pink and blue boxes, respectively, in Figure 3). The two lists of proteins were used for STRING analysis, which produced two interaction networks (Supplementary Figures 3A,B). The two groups of proteins can be sub-grouped into several smaller patterns by the time-resolved mRNA expression. Therefore, we identified the five sub-patterns with the genes in the pink and blue boxes (Figure 3 and Supplementary Table 7). In detail, 343 genes, downregulated in protein level (P_D), were sub-grouped into two patterns of 187 and 156 genes in P_D + R_D and P_D + R_U, respectively, whereas 311 genes, upregulated in protein level (P_U), were sub-grouped into three patterns of 64, 159, and 88 genes in P_U + R_EarlyU, P_U + R_LateU, and P_U + R_D, respectively. Among the five sub-patterns, three sub-patterns of P_U + R_EarlyU, P_U + R_LateU, and P_D + R_D showed similar expression patterns in both mRNAs and proteins, and other patterns of P_U + R_D and P_D + R_U) showed opposite expression patterns (Figure 3). Although both P_U + R_EarlyU and P_U + R_LateU showed the increased proteins and mRNAs levels in P-activated conditions, P_U + R_EarlyU showed the upregulation of mRNA in early time points of 0–15 min, in contrast, P_U + R_LateU showed the upregulation of mRNA in late time points of 30–120 min.

We then profiled the temporal fold changes of mRNAs and proteins between P-activated and control *Xoo* cells (Figure 3). As the proteome datasets do not include protein levels in time points of 5, 10, 15, and 45 min, in which the protein levels were extrapolated based on the protein levels at other time points using non-linear regression.

#### Flagella and Chemotaxis-Related Genes

Flagella and chemotaxis-related genes encode more than 40 proteins, including structural components and assembly factors of flagellar hook-basal body and filament and chemotaxis proteins (Mukherjee and Kearns, 2014). In the time-supervised hierarchical clustering, two dominant clusters of upregulated and downregulated proteins, pink and blue boxes, respectively, were identified (Figure 3). The pink cluster of 311 proteins included 21 (53%) of the 40 genes related to the flagellar assembly pathway, of which 20 genes were included in the red cluster (Supplementary Table 7).

**FIGURE 4:**
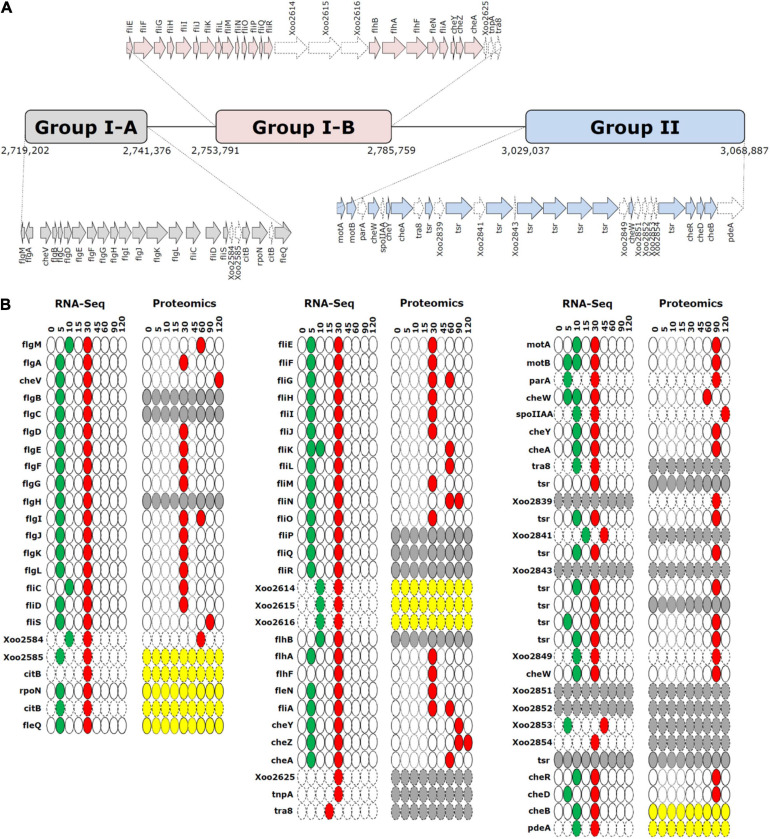
Gene clusters and time-resolved expression patterns of cell motility-related genes. **(A)** Gene clusters of flagellar biosynthesis-related genes (groups I-A and I-B) and chemotaxis genes (group II). **(B)** Time-resolved mRNA and protein expression levels of genes in groups I-A, I-B, and II. The downregulated and upregulation peaks are shown in green and red, respectively. The yellow and gray ovals indicate unaltered and undetected expression levels. The thin-bordered oval (at 5, 10, 15, and 45 min) for the proteome data is to ensure consistency with the pictorial format of RNA-Seq data. The dotted arrows and ovals indicate genes that are not directly related to flagellar biosynthesis and chemotaxis. Time is expressed in min.

We grouped the flagella and chemotaxis-related genes into three gene clusters, i.e., groups I-A, I-B, and II, based on their positions in the genome (Figure 4A). Genes in cluster I-A and I-B were associated with flagellar machinery, such as flagellar basal body hook and type III secretion system (T3SS), whereas cluster II included chemotaxis-related genes.

Superimposition of transcriptome and proteome data of cell motility related genes revealed clearly superimposed upregulated peaks at 30 min (Figure 2A). The transcriptome data revealed that most flagella and chemotaxis-related genes were regulated in a similar pattern, i.e., genes in all clusters of I-A, I-B, and II were downregulated at 5 min and upregulated at 30 min. However, the proteome data presented a different expression pattern between clusters I-A and I-B and cluster II. Proteins in clusters I-A and I-B were upregulated at 30 min, consistent with the transcriptome data. But proteins in cluster II were upregulated at 90 min, exhibiting a delay of 1 h when compared with the transcriptome data (Supplementary Figure 4).

#### Inorganic Ion Transport and Metabolism Genes

**FIGURE 5:**
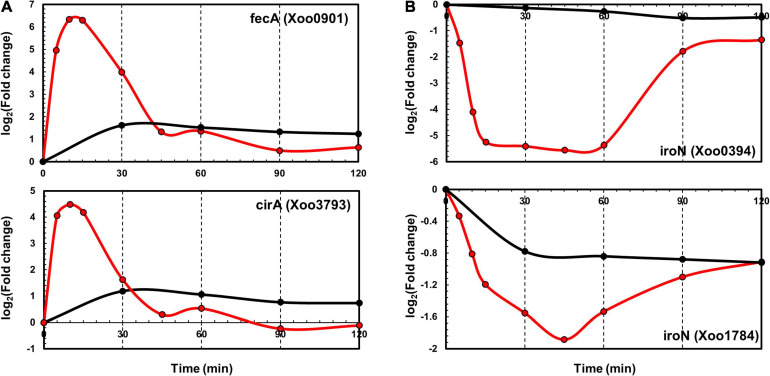
Time-resolved mRNA and protein levels of iron transport-related genes. **(A)** Time-resolved mRNA (red) and protein (black) expression levels of *FecA* and *CirA* genes. **(B)** Time-resolved mRNA (red) and protein (black) expression level of *IroN* genes. The *Y*-axis represents log_2_(fold-change).

TonB-dependent receptors (TBDRs) are bacterial outer membrane proteins that bind and transport ferric chelates of siderophores. Several annotated TBDR genes have been identified in the *Xoo* KACC10331 genome, including *IroN*, *FyuA*, *FecA*, *BtuB*, *FhuA*, *CirA*, and *FepA*. Of these, *FecA* (*Xoo0901*) and *CirA* (*Xoo3793*) were upregulated and *IroN* genes (*Xoo0394* and *Xoo1784*) were downregulated in both the transcriptome and proteome data. In the proteome data, *FecA* (*Xoo0901*) and *CirA* (*Xoo3793*) upregulation peaked at 30 min (Figure 5A). *IroN* genes that were downregulated at the transcript level were also downregulated at the protein level (Figure 5B). The expression levels of other paralogs of *FecA* and *CirA* genes were comparable with those in the control in the transcriptome as well as the proteome data (Supplementary Figure 5), indicating that these genes could respond to different pathogenic signals—which were missing in the *in vitro* system—or be pseudogenes.

Phosphate uptake genes, i.e., *OprO*, *PhoX*, *PstSCAB*, and *PhoU*, were upregulated in the transcriptome (up to 16-fold) at 5–10 min. The proteome data of OprO, PhoX, PstSCAB, and PhoU revealed upregulation up to three-fold at 30 min (Supplementary Figure 6).

#### Expression and Secretion of Effectors

**FIGURE 6:**
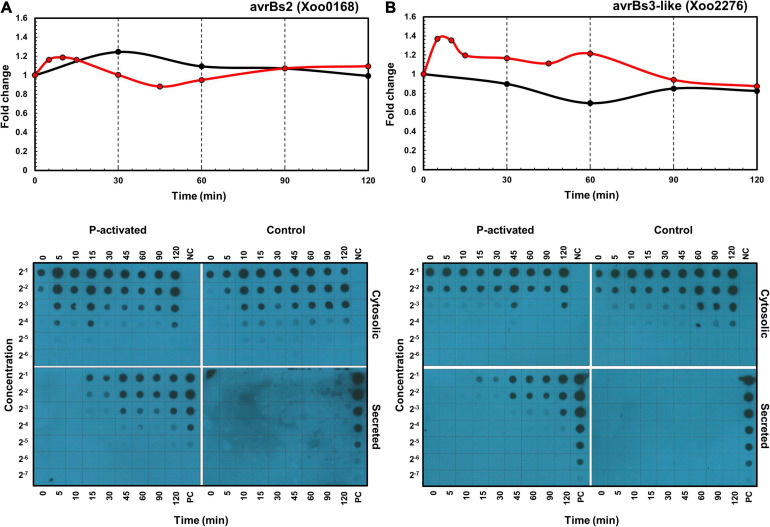
Time-resolved mRNA and protein expression and secretion of effector proteins. The mRNA (red) and protein (black) expression levels of *XoAvrBs2* **(A)** and *XoAvrBs3* **(B)** genes in the transcriptome and proteome data (above) and the cytosolic and secreted XoAvrBs2 **(A)** and XoAvrBs3 **(B)** proteins in the dot blot data (below). NC, negative control; PC, positive control.

In addition to the transcriptome and proteome data, the expression and secretion of effectors XoAvrBs2 (*Xoo0168*) and XoAvrBs3 (*Xoo2276*) were assessed using dot blots of *Xoo* cells transformed with the TAP-tagged effector genes (Figure 6). In protein levels, proteome data provide only cellular levels after 30 min and dot blot data provide cellular levels and secreted levels throughout the whole time. The cellular protein levels after 30 min were available in both proteome and dot blot data and were similar in both data. The secreted protein levels were dramatically upregulated from 15 min.

In detail, the expression of *XoAvrBs2* transcript was upregulated from 5 to 30 min, while that of the XoAvrBs2 protein was upregulated at 30 min in the transcriptome and proteome data. In the dot blots, the levels of TAP-tagged XoAvrBs2 were upregulated by four-fold at 5–15 min and the secretion of XoAvrBs2 was detected as early as 15 min. The expression of *XoAvrBs3* transcript was upregulated and peaked from 5 to 15 min, whereas that of XoAvrBs3 protein was maintained at almost the same—or slightly lower levels—at 60 min in the transcriptome and proteome data. Dot blots revealed the secretion of XoAvrBs3 from 15 min.

## Discussion

Plant pathogens could exhibit complex responses to the initial interactions with the host under varying biotic and abiotic conditions at the site of infection. The varied immediate response of the plant pathogen is important for successful infection. In this study, we analyzed the immediate time-resolved response of *Xoo* cells from the initial interaction with rice in terms of gene expression at both the mRNA and protein levels using an *in vitro* assay system, wherein RLX mimicked the damaged rice leaf tissue (Scheme 1). The *in vitro* assay system contains the host rice components from RLX and provides us the homogenous and synchronized *Xoo* cells upon the initial interaction to rice, which were not possible in the previous assay systems of minimum media-using *in vitro* system (Tsuge et al., 2002) and traditional *in vivo* system (Roden et al., 2004). However, the *in vitro* assay system in this study is still one of the nature mimicking assay systems.

Of all the predicted open reading frames in *Xoo*, we quantified approximately 83% and 47% of the mRNAs and proteins, respectively, in a time-dependent manner. A good correspondence of overall expression pattern was observed between the mRNA and protein levels of genes. The expression pattern was more synchronized for genes closely located in the genome (Figure 4 and Supplementary Figure 4); this could be attributed to the polycistronic gene structure in bacteria. With respect to the rapidity of pathogenic gene expression upon interaction with the host, both transcriptional and translational machineries responded immediately to the interaction, at the earliest assessed time points of 5 and 30 min in the transcriptome and proteome data, respectively. The transient expression patterns in *Xoo* accorded well with the initial surge and later declination of gene expression in bacterial pathogens upon environmental pathogenic signals (Shin et al., 2006). The secretion of effectors was also found at 15 min in dot blots. Both mRNAs and proteins presented the greatest variation in their levels during the initial 30 min.

The *Xoo* genes exhibiting the most rapid responses to the initial interaction with RLX, included genes associated with cell motility and inorganic ion uptake, and genes coding for effector molecules. All these three functional categories of genes are closely related to the early stages of pathogenesis. The genes associated with cell motility could be responsible for the migration and accumulation of the *Xoo* cells at the site of infection via the damaged xylem tissues or exposed hydathodes in rice. As inorganic ions, such as iron and phosphate ions, function as essential cofactors in all living organisms, bacterial pathogens and host rice cells compete to obtain and secure the limited resources available. Effector molecules are more directly related to pathogenesis and are injected by the *Xoo* into the rice cells via pili-like T3SS; these molecules modulate the immune system of rice.

The earliest available time-point for comparing the gene expressions in terms of mRNA and protein levels was 30 min after the RLX treatment. At 30 min, 290 mRNAs were upregulated by more than two-fold in the transcriptome, compared with 93 proteins in the proteome (Supplementary Table 4), and the average fold change in the expression of the upregulated genes in the transcriptome and proteome was similar, i.e., 3.3-fold and 3.4-fold, respectively (Supplementary Table 5). Even though we consider the higher coverage of quantified genes in transcriptome than in proteome, the number of upregulated transcripts is much higher than that of the proteins at 30 min, which indicates that not all the upregulated mRNAs are simultaneously translated to proteins.

For some genes, the mRNA and protein expression peaks were observed at varying time points. The difference in the gene expression in terms of the mRNA and protein levels suggests the existence of a fine-tuning translational regulation step in bacterial pathogenesis, which could help adjust the expression of the early-responsive genes under varying biotic and abiotic environmental conditions.

Genes related to flagella and chemotaxis are clustered in the bacterial genome. In *Xoo*, three clusters are observed, i.e., I-A, I-B, and II. On superimposing the mRNA and protein levels in time, the expression of mRNAs and proteins peaked at 30 min for genes in the clusters I-A and I-B, the expression of mRNAs and proteins peaked at 30 and 90 min, respectively, for genes in cluster II. A translational regulation step might be involved that determines the time for the translation of specific mRNAs—coded by flagella and chemotaxis-related genes—into proteins depending on the priority of each gene.

The hierarchy of the expression of flagella and chemotaxis-related genes has been extensively studied in the transcriptome of *Escherichia coli*, where sigma and anti-sigma factors are known to be the key transcriptional regulators (Typas and Sourjik, 2015; Ni et al., 2017). The overall organization of flagella and chemotaxis-related genes is different between *E. coli* and *Xoo* (Supplementary Figure 7). In *Xoo*, the cluster of *fliE-R* genes (cluster I-B) is positioned just downstream to the *flgB-L* genes (cluster I-A), whereas in *E. coli*, chemotaxis genes are present between the two. *flhDC* genes do not have any orthologs in *Xoo*, and a different transcriptional regulator, i.e., the *fleQ* gene, is present. Based on the nomenclature used in *E. coli*, several *Xoo* genes classified as Class III genes are located at different positions and their expression regulation is also different. In *Xoo*, ribosomes or other translation factors might recognize certain unknown priority signals in mRNA transcripts for the translational regulation.

The immediate upregulation of inorganic ion uptake genes, especially iron uptake genes, may aid *Xoo* cells to obtain the essential cofactor ions, when they are competition with the host cells (Liu et al., 2020). The iron ion is essential for photosynthesis and respiration and needed by many redox enzymes for most organisms on Earth. Iron uptake genes play a key role in pathogenicity during the early stages of host-pathogen interactions (Garau et al., 2004). Pathogens commonly use iron chelating molecules or siderophores to derive this scarce inorganic cofactor from the hosts. The leakage of iron ions from damaged leaf tissues might serve as an important signal of initiating infection and might provide an opportunity to secure essential iron ions for the *Xoo* cells.

In comparison with the flagella and chemotaxis-related genes that are closely clustered in the genome and exhibit coordinated expression levels, the ion uptake genes are dispersed across the genome and are independently expressed (Supplementary Figure 8). The separation of these genes facilitates independent regulation. Compared with the flagella and chemotaxis-related genes, the inorganic ion uptake genes exhibited great variation in the mRNA levels but similar protein expression levels (Figure 2).

Effectors are key molecules for pathogenicity that modulate the host immune responses after infection. In *Xanthomonas oryzae* pv. *oryzicola*, AvrBs2 suppresses host immunity and promotes disease development (Ullah et al., 1998). In *Xanthomonas campestris* pv. *vesicatoria*, AvrBs3 activates the expression of plant immunity genes by working as a transcription activator-like (TAL) effector (Kay et al., 2009). The secretion of effectors XoAvrBs2 and XoAvrBs3 through T3SS was confirmed upon interaction with RLX (Kim et al., 2011, 2013).

The protein expression and secretion of the *Xoo* effectors XoAvrBs2 and XoAvrBs3 were assessed using dot blots, which enabled the monitoring of effector proteins at the early stages of the initial interaction, i.e., within 30 min of RLX treatment. The secretion of effectors was observed from 15 min after application of the pathogenic stimulus. In the P-activated proteome data, the expression of effector proteins was maintained at a similar level to that at 0 min; this may be attributed to the similar protein synthesis and secretion rates.

The effector genes are also dispersed across the genome, like the ion uptake genes (Supplementary Figure 9). Interestingly, transposase genes are located close to the effector genes; these may aid the effector genes to transpose through the bacterial genome and plasmids. The cellular expression level of XoAvrBs2 was upregulated at 5 min in mRNAs and proteins, and its secretion was detected at 15 min (Figure 6). Although the cellular protein level was upregulated by approximately 20% in the proteome at 30 min, the dot blot showed an upregulation of four-fold at 5–15 min. The secreted XoAvrBs2 exhibited a 16-fold increase at 120 min. In case of XoAvrBs3, the cellular protein level was not upregulated or maintained at a similar level in both proteome and dot blots data, whereas the secretion was detected from 15 min and increased up to 16-fold at 120 min. The experimental methods for proteome and dot blots analysis are different (Kim et al., 2011, 2013). For the dot blots analysis, TAP-tagged *XoAvrBs2* and *XoAvrBs3* genes were introduced into *Xoo* cells via the plasmid having the endogenous promotor and expressed from the plasmid. In the proteome analysis, XoAvrBs2 and XoAvrBs3 proteins were expressed from the endogenous *XoAvrBs2* and *XoAvrBs3* genes in the *Xoo* genome. Therefore, the expression levels from both data could be varied.

In addition to the effectors transported via T3SS, plant cell wall degrading enzymes are transported via T2SS. Genes like protease *HtrA* (*Xoo0059*), cellulase *Egl* (*Xoo0281*), cellulase *CelS* (*Xoo1076*), and xylanase *XynB* (*Xoo1371*) were upregulated in transcriptome and Egl (*Xoo0281*) was upregulated in proteome (Supplementary Figure 10). Genes related to the nitrogen metabolism of P_II_ signal transduction (Huergo et al., 2013) such as *GlnB* (*Xoo0213*), *GlnB* (*Xoo4487*), *PtsN* (*Xoo1282*), and DraG (*Xoo1743*) were upregulated at 5–60 min and PtsN (*Xoo1282*) was slightly upregulated at 30 min (Supplementary Figure 11).

In the time-supervised hierarchical clustering of both transcriptome and proteome, some genes were differently regulated in mRNA and protein levels such as P_D + R_U and P_U + R_D (Figure 3). The further study is necessary to understand the pairwise combined analysis between mRNAs and proteins in time. The analysis of gene expression and regulation in this study is mainly carried out by comparing the fold change of mRNAs and proteins of each gene in time, without considering the absolute amount of each component. For example, very low expression levels in some genes could provide statistical misinterpretations in the comparison of fold changes in time. In addition, proteome data do not have protein expression levels before 30 min, compared to mRNA levels, when many pathogenicity-related genes were regulated transcriptionally in the *in vitro* assay system. The early proteome data before 30 min would provide valuable information to understand the pathogen-host interactions at early stage.

**FIGURE 7:**
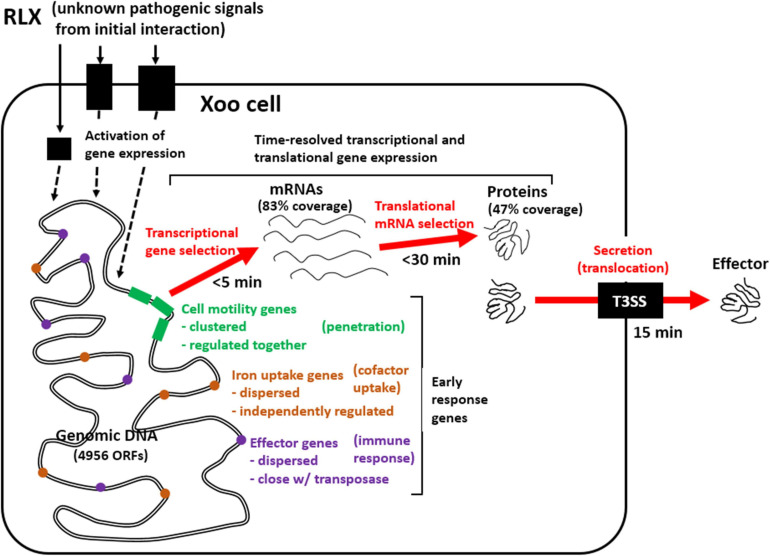
Schematic representation of genome-wide pathogenic gene expression and effector secretion via *in vitro* assay system. Early response *Xoo* genes from the initial interaction with RLX include genes related to cell motility, iron uptake, and effector, of which expression are upregulated as early as 5 min in mRNAs and 30 min in proteins and effector secretion is found since 15 min.

Gene expression involves sequential transcription and translation. In bacteria, with respect to post-transcriptional and post-translational modification, mRNAs without a cap at 5′ end and a poly A tail at 3′ end have a short half-life—as short as few minutes—and proteins undergo only limited post-translational modifications. The limited post-translational modifications in bacterial proteins impose a pressure on a nascent protein from the ribosome to take a functional form immediately. The present study using the *in vitro* assay system revealed that genes related to cell motility and inorganic ion uptake, and genes coding for effector molecules of *Xoo* are the first to respond to the initial interactions with RLX, and play an essential role in the following developments of *Xoo* pathogenesis (Figure 7): (1) invasion of the *Xoo* cells into the rice leaf tissues, (2) securing the limited cofactors, and (3) modulating the immune responses of the host to favor pathogenesis. This combined analysis of the time-resolved transcriptome and proteome of *Xoo* during the initial interaction with rice tissues provides valuable insights into the pathogenic mechanism of *Xoo*.

## Materials and Methods

### Bacterial Strain and Culture Conditions

*Xanthomonas oryzae* pv. *oryzae* (*Xoo*) strain KACC10331, consisting of 4,941,439 nucleotides and 4,733 open reading frames, without any apparent autonomous plasmids, was obtained from the Korean Agricultural Collection Center (KACC) (Lee et al., 2005). The bacteria were cultured in nutrient broth (Difco, Detroit, MI, United States) or Yeast Glucose Cm Agar (YGC) [1% yeast extract, 2% D-(+)-glucose, 2% CaCO_3_, and 1.5% agar] at 28°C.

### Construction of Expression Vector and Transformation of *Xoo*

The effector genes *XoAvrBs2* (*Xoo0168*) and *XoAvrBs3*—including the promoter region (from −149 to −750 bp to the start codon of the respective gene)—were amplified by PCR and ligated into the pGEM-T Easy Vector (Promega). The cloned sequences were verified and then digested with *Kpn*I and *Sac*I, and the products were ligated into the pHM1-XTAP-T_gap_ vector. The recombinant vectors were purified and introduced into *Xoo* strain KACC10331 by electroporation, using Gene Pulser II (Bio-Rad, Hercules, CA, United States) with a 0.2 cm-gap cuvette at 2.5 kV cm^–1^. *Xoo* cells were then diluted immediately with 1 mL Super Optimal Broth (SOC) medium and incubated at 28°C with agitation for 2 h. Cells were then recovered from the culture medium and plated on nutrient broth agar plates containing 50 μL mL^–1^ spectinomycin and incubated at 28°C for 4 days.

The transformants were cultured in 100 mL of nutrient broth up to the mid-exponential phase (OD_600_ = 0.5). Cells were harvested by centrifuging 1 mL of the cell culture at 12,000 rpm and 4°C for 5 min. Harvested cells were washed once with phosphate-buffered saline (PBS) at pH 7.2, resuspended in 200 μL PBS, and then sonicated. Protein samples were serially diluted using 2 M urea in PBS in 96-well plates and then transferred to a polyvinylidene difluoride membrane (PVDF; 0.2 μm, Bio-Rad) using a 96-well vacuum dot-blotter (Bio-Rad). The membrane was then washed thrice with PBS, blocked with 5% skim milk for 30 min, and subjected to a one-step immuno-affinity reaction using the rabbit peroxidase-anti-peroxidase soluble complex antibody (Sigma-Aldrich, St. Louis, United States). The membrane was developed, and bound antibodies were detected by chemiluminescence.

### Treatment of Rice Leaf Extract for Proteome Analysis

*Oryza sativa* L. cv. Milyang 23, a *Xoo*-susceptible rice cultivar, was used for performing proteome analysis. Rice plants were grown in a paddy field at Jeonju in South Korea (35°49′52.0″N 127°03′55.6″E) until panicle initiation (approximately 8–9 weeks). Forty clumped rice leaves were harvested and homogenized with liquid nitrogen using a mortar and pestle. One-gram aliquots of the resulting RLX were transferred to Eppendorf tubes and stored at −80°C. *Xoo* was cultured (100 mL) in nutrient broth up to the mid-exponential phase (OD_600_ = 0.5) in a shaking incubator at 28°C and 200 rpm, and RLX (2 g) was then added to the culture medium. The culture (100 mL) was subjected to sequential filtration through a gauze, 40-μm nylon cell strainer (FALON, New York, United States), and 5-μm syringe filter (Sartorius, Germany) to remove RLX (0, 30, 60, 90, and 120 min after RLX addition). The filtered culture (100 mL) was centrifuged at 10,000 × *g* and 4°C for 10 min. Duplicate samples were obtained for each time point from two independent experiments.

### Sample Preparation for Proteome Analysis

The harvested samples were lysed in a lysis buffer containing 9 M urea prepared in 20 mM HEPES (pH 7.5), supplemented with protease inhibitor cocktail (Complete mini, Roche) and phosphatase inhibitor (PhosSTOP, Sigma-Aldrich) and sonicated on ice. The exact amount of proteins in each sample was determined using the bicinchoninic acid assay. Protein integrity was confirmed by SDS-PAGE and 200 μg of protein from each sample was used for analysis. The disulfide bonds were reduced by treatment with 10 mM dithiothreitol for 1 h, and incubation with 30 mM iodoacetamide (30 min in the dark) was performed to alkylate free sulfhydryl functional groups. Samples were diluted with triethylammonium bicarbonate buffer (pH 8.0) in a manner such that the final urea concentration was 1.5 M. Proteins were digested using MS grade trypsin (Thermo Fisher Scientific) at a protein to enzyme ratio of 50:1 for 12 h at 37°C. The reaction was quenched by lowering the sample pH (<3) using trifluoroacetic acid. The obtained peptides were desalted using a C18 spin column (Harvard) to remove salts and other contaminants, and the purified peptides were dried. Then, they were isotopically labeled using the 10-plex tandem mass tag (TMT, Thermo Fisher Scientific), as per the manufacturer’s protocol. The reaction was allowed to continue for 2 h at room temperature and TMT-labeled samples were subsequently dried in a SpeedVac concentrator. Chemical labeling with TMT was confirmed by liquid chromatography-tandem mass spectrometry (LC-MS/MS), and the samples were pooled and fractionated using a basic reverse phase liquid chromatography (RPLC) system. The pooled TMT-labeled peptide mixture was resuspended in 10 mM ammonium formate and fractionated into 12 fractions using a C18 column (C_18_, 5 μm pore size, 4.6 mm × 250 mm, XBridge, Waters). The fractionated peptides were dried and stored at −80°C until LC-MS/MS analysis.

### LC-MS/MS and Proteome Data Analysis

Each fractionated peptide sample was analyzed using an Orbitrap Fusion^TM^ Lumos^TM^ Tribrid^TM^ Mass Spectrometer coupled with the Easy-nLC 1200 nano-flow liquid chromatography system (Thermo Fisher Scientific). The dried peptides were reconstituted using 0.1% formic acid and loaded on a C18 trap column. Peptides were resolved using a linear gradient solvent B (0.1% formic acid in 95% acetonitrile) and analyzed by high resolution mass spectrometry in the data-dependent acquisition mode. MS1 and MS2 were acquired for the precursor and the peptide fragmentation ions, respectively. MS1 scans were measured at a resolution of 120,000 and an *m/z* of 200. MS2 scans were acquired following the fragmentation of precursor ions by high-energy collisional dissociation (HCD) and were detected at a mass resolution of 50,000 and an *m/z* of 200. Dynamic exclusion was used to reduce redundant fragmentation of the same ions. The obtained mass spectrometry data were analyzed using the MaxQuant software (Tyanova et al., 2016a). Raw MS data were searched against the *Xoo* proteome in UniProt database. Carbamidomethylation of cysteine and 10-plex TMT modification of lysine and N-terminals were set as static modifications, whereas oxidation of methionine was set as a variable modification. False discovery rates at the levels of protein and peptide-spectrum matches were set at 0.01. The raw MS data and MaxQuant search results have been submitted to ProteomeXchange (project accession: PXD020135, reviewer access with username: reviewer34070@ebi.ac.uk and password: mzn76I1O).

The contaminant and reverse identified proteins were removed from the MaxQuant data. Proteins identified in both replicates were pooled for quantile normalization. The normalized values for the replicates were subjected to supervised hierarchical clustering and principal component analysis, using Perseus (Tyanova et al., 2016b), and the results were depicted in the form of a multi-scatter plot.

### RNA-Seq and Transcriptome Data Analysis

In addition to the previously obtained RNA-Seq data for P-activated and control *Xoo* cells, RNA-Seq data at 0, 90, and 120 min were obtained to correspond with the proteome data at these time points. RNA-Seq and data analysis were performed as previously described (Kim et al., 2016). Briefly, total RNA from samples was used to generate sequencing libraries, from which ribosomal RNA was removed using MICROBExpress Bacterial mRNA Enrichment Kit (Ambion, Austin, TX, United States), and enriched mRNA was prepared using Illumina TruSeq RNA Sample Preparation Kit (Illumina, San Diego, CA, United States). The RNA obtained after fragmentation was used to generate cDNA fragments, which were sequenced using Illumina Genome Analyzer IIx and mapped to the reference genome sequence^1^ using CLC Genomics Workbench 4.0 (CLC bio, Aarhus, Denmark). Relative transcript abundance was calculated based on the number of reads per kilobase per million mapped sequence reads (RPKM).

### Analysis of Time-Resolved Continuous mRNA and Protein Expression

The RPKM values of the mRNAs in the transcriptome and TMT intensities of the proteins in the proteome corresponded to the observed expression level of each gene at a given time point. To facilitate the comparison of gene expression levels, the observed expression level at each time point was converted to fold change in gene expression, by dividing the expression level at a given time point by the initial expression level (0 min) of the same gene. The fold change in the time-resolved expression levels of a given gene during the 2 h following the RLX treatment was fitted to a curve and analyzed using non-linear regression by GraphPad Prism (version 3.02 for Windows, GraphPad Software, San Diego, CA, United States)^2^, and the continuous time-dependent changes in the mRNA and protein expression levels were determined using the fitted curve.

For the simple comparison of the mRNA and protein expression levels in P-activated *Xoo* cells, the expression levels were corrected by comparing with those in the control cells at each time point. The fold change in mRNA and protein expression in P-activated *Xoo* cells at a given time point was divided by that of the control cells at the same time point. The resulting control-corrected fold change values of mRNAs and proteins were considered as the relative gene expression levels of mRNAs and proteins at each time point.

### Comparison of Expression Patterns of mRNAs and Proteins

For the comparison of mRNA and protein datasets, we first generated the integrative expression matrix by merging the two omics expression matrices. In detail, each matrix was processed via following steps. For proteome expression matrix, two TMT expression matrices were log2-quantile normalized and *z*-transformed independently. For transcriptome expression matrix, both RPKM matrices of dataset set1 for 0, 5, 10, 15, 30, 45, and 60 min and dataset set2 for 0, 90, and 120 min were log2-quantile normalized. The values in set2 were normalized by that at set2 0 min, centered by that at set1 0 min, and merged into set1 sequentially. The merged RPKM matrix was z-transformed. Among the detected protein-coding genes in proteome and transcriptome, we selected genes with observation of more than 3 times in both omics matrices for further analysis and made the integrative expression matrix of TMT set1, TMT set2, and merged RPKM. Then, we performed the hierarchical clustering analysis (distance: average; method: Pearson’s method) to investigate the gene expression patterns.

Based on the hierarchical clustering result, we selected the optimal sub-pattern number for upregulated and downregulated proteome patterns (three sub-groups for upregulated genes and two sub-groups for downregulated genes) and performed the k-means clustering to each proteome pattern.

### STRING Map Analysis

The list of proteins in the upregulated and downregulated patterns from the hierarchical clusters was uploaded on the STRING database^3^ to analyze the protein interaction maps. The clusters of proteins in the upregulated and downregulated classes, which show the similar time-resolved expression patterns, were analyzed, and the list including the gene names with the selected organism was inputted in the multiple proteins search setting. The number of nodes and edges were automatically calculated based on *Xoo* genes with a PPI enrichment p-value of 2.09E-9. Figures were downloaded in the PNG file format for visualization.

## Data Availability Statement

The datasets presented in this study can be found in online repositories. The names of the repositories and accession numbers can be found below: ProteomeXchange (Project 541 accession: PXD020135) and the RNA-Seq data were deposited in NCBI GEO (Gene 543 Expression Omnibus) with the accession numbers GSE154542 and GSE61607.

## Author Contributions

SK, WJ, JP, M-SK, J-GK, and L-WK: investigation, writing, and methodology. J-GK and L-WK: funding acquisition. M-SK, J-GK, and L-WK: supervision. All authors have read and agreed to the published version of the manuscript.

## Conflict of Interest

The authors declare that the research was conducted in the absence of any commercial or financial relationships that could be construed as a potential conflict of interest.

## Footnotes

1 http://www.ncbi.nlm.nih.gov/nuccore/58579623?report=fasta

2 www.graphpad.com

3 https://string-db.org/

## Supplementary Material

Supplementary Figure 1 Detailed analysis of the proteome data of pathogenicity-activated *Xoo* cells **(A)** Number of proteins identified from the high-resolution mass spectrometry-based quantitative proteomic analysis and **(B)** coverage percentage of the protein sequence of the identified proteins (median sequence coverage was ∼24%). **(C)** Number of identified peptides and peptide-spectrum matches.

Supplementary Figure 2 Comparative analysis of the proteome of the samples **(A)** Quantile normalization box plot. The quantitative proteomic values were normalized using quantile normalization. **(B)** Multi-scatter plot for analyzing the correlation between samples. Pairwise comparisons of protein expression levels in all samples are presented as a multi-scatter plot. Pearson’s correlation coefficients of 0.98–0.99 were obtained. **(C)** Principal component analysis of *Xoo* proteins revealed close relationships among the proteomes of all controls and changes in proteome concentrations upon RLX treatment.

Supplementary Figure 3 Analysis and comparison of gene expression patterns in the datasets. The STRING maps (Benjamin-Hochberg at FDR 0.05) of selected cluster profile patterns with **(A)** downregulated (blue box) and **(B)** upregulated (red box) genes are shown.

Supplementary Figure 4 Comparison of time-resolved mRNA and protein levels of cell motility-related genes. **(A)** Time-resolved mRNA levels of flagellar biosynthesis and chemotaxis-related genes for pathogenicity-activated *Xoo* cells. All genes in groups I-A, I-B, and II exhibited the lowest expression level at 5 min and the highest expression level at 30 min. **(B)** Time-resolved protein levels of flagellar biosynthesis and chemotaxis-related genes in pathogenicity-activated *Xoo* cells. Genes in groups I-A and I-B exhibited the highest expression level at 30 min, whereas those in group II exhibited the highest expression at 90 min.

Supplementary Figure 5 Time-resolved mRNA and protein expression of iron uptake-related genes. Time-resolved mRNA (red) and protein (black) expression levels of iron uptake-related genes **(A)** *FecA* and **(B)** *CirA*. The *Y*-axis represents log_2_(fold change).

Supplementary Figure 6 Gene cluster and time-resolved mRNA and protein expression levels of phosphate uptake-related genes. **(A)** Gene cluster of the phosphate uptake regulation genes (*OprO-PhoX-PstSCAB-PhoU*). **(B)** Time-resolved mRNA and protein expression levels of phosphate uptake-related genes *OprO*, *PhoX*, *PstSCAB*, and *PhoU.* The line colors correspond to those of the arrows in **(A)**.

Supplementary Figure 7 Gene clusters of cell motility-related genes in *E. coli* and *Xoo* and time-resolved mRNA and protein expressions of *Xoo* genes. Gene cluster of cell motility-related genes of **(A)** *E. coli* and **(B)** *Xoo*. **(C)** Time-resolved mRNA and protein expression of cell motility-related genes in *Xoo*. The cell motility-related genes in *E. coli* are classified as class I (red), II (yellow), III (blue), and II + III (green). The ortholog genes in *Xoo* are indicated using the same color. For the proteome data, * indicates the expression peak at 30 min; **, between 30 and 90 min; ***, at 90 min. The *Y*-axis represents log_2_(fold change).

Supplementary Figure 8 Gene clusters and time-resolved mRNA and protein expressions of iron uptake-related genes in *Xoo*. **(A)** Gene cluster of iron uptake-related genes, labeled in blue. **(B)** Time-resolved mRNA and protein expression levels of iron uptake genes. Low to high expression is indicated by a change in color from blue to red. Black cells indicate undetected expression.

Supplementary Figure 9 Gene clusters and time-resolved mRNA and protein expressions of genes coding for effector molecules. **(A)** Gene cluster of effector genes, indicated in red. **(B)** Time-resolved mRNA and protein expressions of effector genes. Genes with available time-resolved proteomic data are labeled with *. Transposase genes are indicated in blue.

Supplementary Figure 10 Time-resolved mRNA and protein expression of plant cell wall degradation-related genes. Genes like *HtrA* (*Xoo0059*), *Egl* (*Xoo0281*), *CelS* (*Xoo1076*), and *XynB* (*Xoo1371*) were upregulated in transcriptome data. In proteome data, Egl (*Xoo0281*) was upregulated by 30% and HtrA (*Xoo0059*) was maintained at a similar level to that at 0 min.

Supplementary Figure 11 Time-resolved mRNA and protein expression of nitrogen metabolism-related genes. Genes like *GlnB* (*Xoo0213*), *GlnB* (*Xoo4487*), *PtsN* (*Xoo1282*), and *DraG* (*Xoo1743*) were upregulated at 5–60 min in transcriptome data. In proteome data, PtsN (*Xoo1282*) was slightly upregulated at 30 min.

Supplementary Table 1 The raw data of time-resolved expression levels of mRNAs and proteins in the transcriptome and proteome of P-activated and control *Xoo* cells. The RPKM values of mRNAs and TMT intensities of proteins were obtained from independent duplicated transcriptome and proteome analyses, respectively, representing the expression levels of mRNAs and proteins of each gene. The gene products that were not identified or measured are indicated by blank cells.

Supplementary Table 2 The zero time-corrected fold change of time-resolved expression levels of mRNAs and proteins in the transcriptome and proteome of P-activated and control *Xoo* cells. The fold change (compared to 0 time) of expression levels is calculated by dividing the expression levels of mRNAs and proteins at a given time point by that at 0 min. Cells are colored based on the expression level; low to high expression is indicated by a change in color from blue to red, with white indicating a fold change of 1.

Supplementary Table 3 The control-corrected fold change of time-resolved expression levels of mRNAs and proteins in the transcriptome and proteome of P-activated *Xoo* cells. For the convenient comparison of the mRNA and protein expression levels, the expression levels of mRNAs and proteins in P-activated *Xoo* cells at each time point was divided by that of the control cells (RLX-untreated *Xoo* cells) at the same time point. The number and percentage of genes exhibiting more than 20%, 50%, and 200% (2-fold) upregulated and downregulation are indicated at the bottom of the table.

Supplementary Table 4 The number of consistently upregulated and downregulated genes for 120 min. The numbers of more than 20%, 50%, and 2-fold upregulated and downregulated genes are measured in *Xoo* cells for the initial 20 min since the first interactions with rice.

Supplementary Table 5 The list of genes having 2-fold upregulated proteins and mRNAs at 30 min. The lists contain genes having more than 2-fold upregulated proteins at 30 min in both duplicate proteome datasets (S6-1), genes having more than 2-fold upregulated proteins at 30 min in the proteome repetition dataset 1 (S6-2) and 2 (S6-3), and genes having more than 2-fold upregulated mRNAs at 30 min in the transcriptome dataset (S6-4).

Supplementary Table 6 The list of genes having 50% downregulated proteins at 30 min in the duplicate proteome datasets. The list contains genes having more than 50% downregulated proteins at 30 min in both proteome datasets.

Supplementary Table 7 The list of selected genes with similar protein expression patterns in the time-supervised hierarchical clustering. The list of genes marked with blue (downregulated) and pink (upregulated) rectangles in Figure 3, which presented distinct synchronized protein expression patterns. Of the 40 flagellum-related KEGG annotated genes, 21 were included in the list, of which 20 genes were upregulated and one gene was downregulated in the proteome.

## Abbreviations

*Xoo*: *Xanthomonas oryzae* pv. *oryzae*
RLX: rice leaf extracts
KACC: Korean Agricultural Collection Center
P-activated: Pathogenicity-activated
LC-MS/MS: liquid chromatography-tandem mass spectrometry
COG: clusters of orthologous groups
RPKM: reads per kilobase per million mapped reads.
