# Supplementary figures and images for: Combined Analysis of the Time-Resolved Transcriptome and Proteome of Plant Pathogen Xanthomonas oryzae pv. oryzae
Source: Front Microbiol. 2021 Jun 2;12:664857. doi: 10.3389/fmicb.2021.664857 (PMC8220824; doi:10.3389/fmicb.2021.664857)

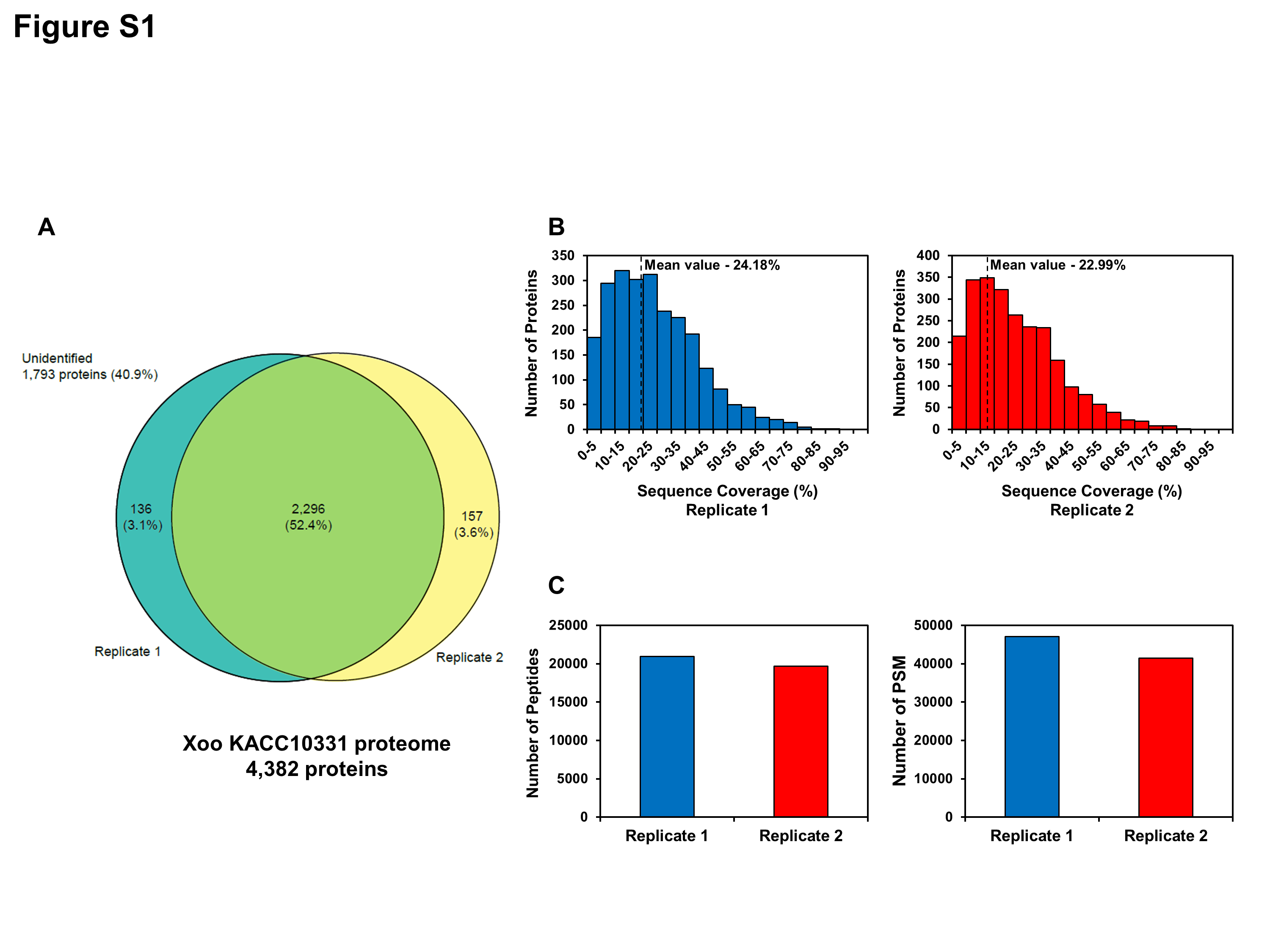

Supplement: Supplementary Figure 1 — Detailed analysis of the proteome data of pathogenicity-activated Xoo cells (A) Number of proteins identified from the high-resolution mass spectrometry-based quantitative proteomic analysis and (B) coverage percentage of the protein sequence of the identified proteins (median sequence coverage was ∼24%). (C) Number of identified peptides and peptide-spectrum matches. [file Image_1.TIF]

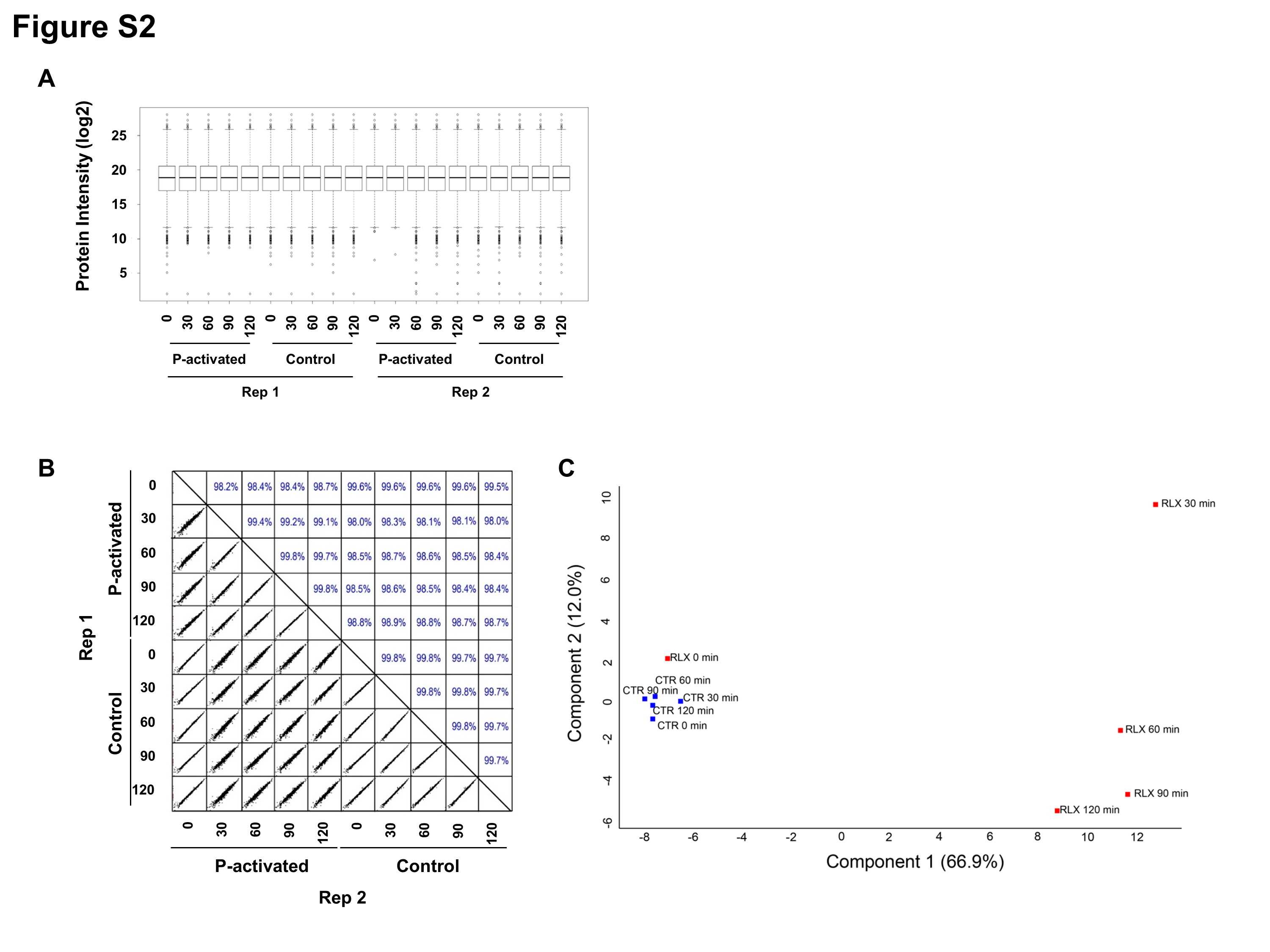

Supplement: Supplementary Figure 2 — Comparative analysis of the proteome of the samples (A) Quantile normalization box plot. The quantitative proteomic values were normalized using quantile normalization. (B) Multi-scatter plot for analyzing the correlation between samples. Pairwise comparisons of protein expression levels in all samples are presented as a multi-scatter plot. Pearson’s correlation coefficients of 0.98–0.99 were obtained. (C) Principal component analysis of Xoo proteins revealed close relationships among the proteomes of all controls and changes in proteome concentrations upon RLX treatment. [file Image_2.TIF]

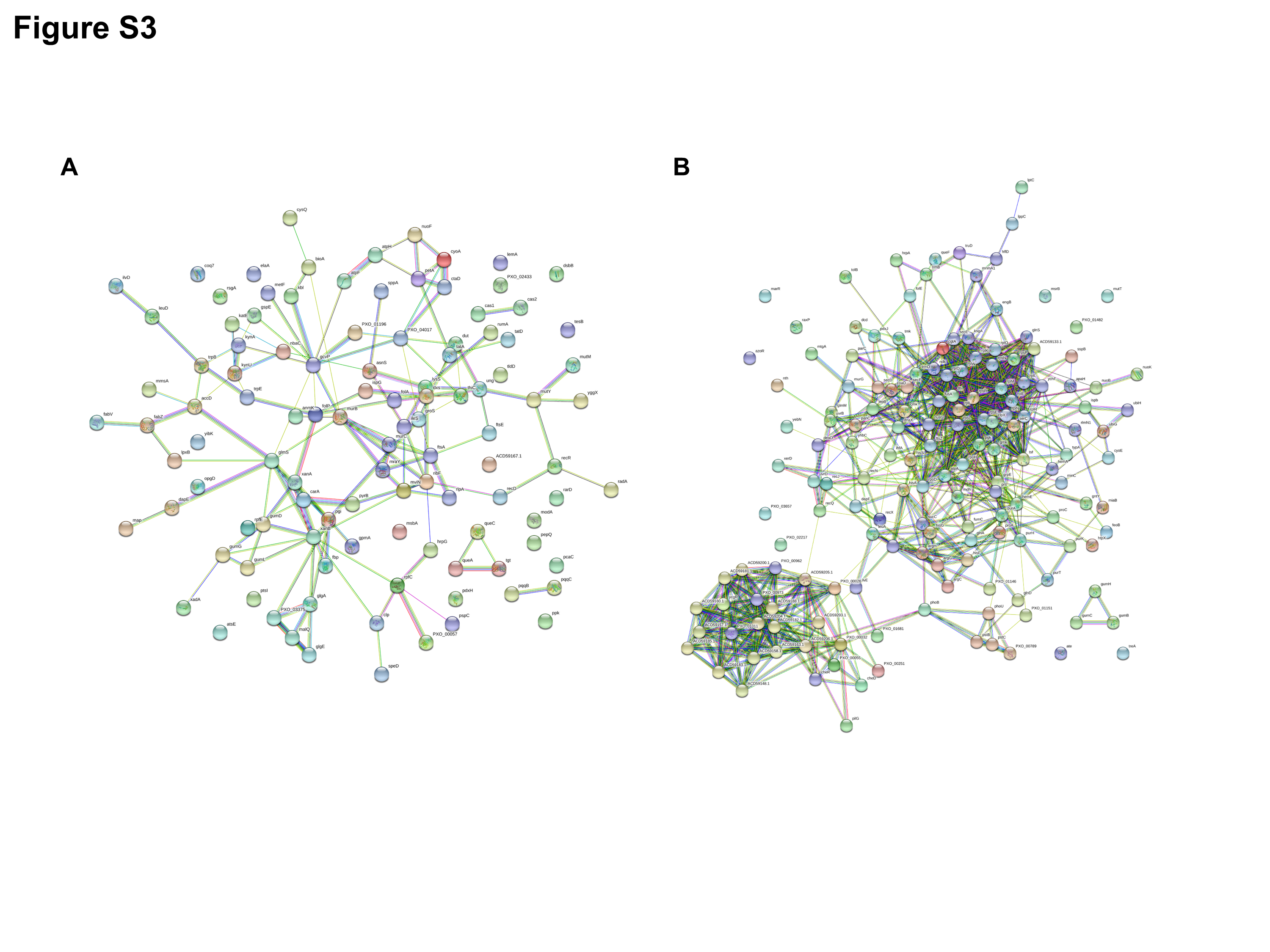

Supplement: Supplementary Figure 3 — Analysis and comparison of gene expression patterns in the datasets. The STRING maps (Benjamin-Hochberg at FDR 0.05) of selected cluster profile patterns with (A) downregulated (blue box) and (B) upregulated (red box) genes are shown. [file Image_3.TIF]

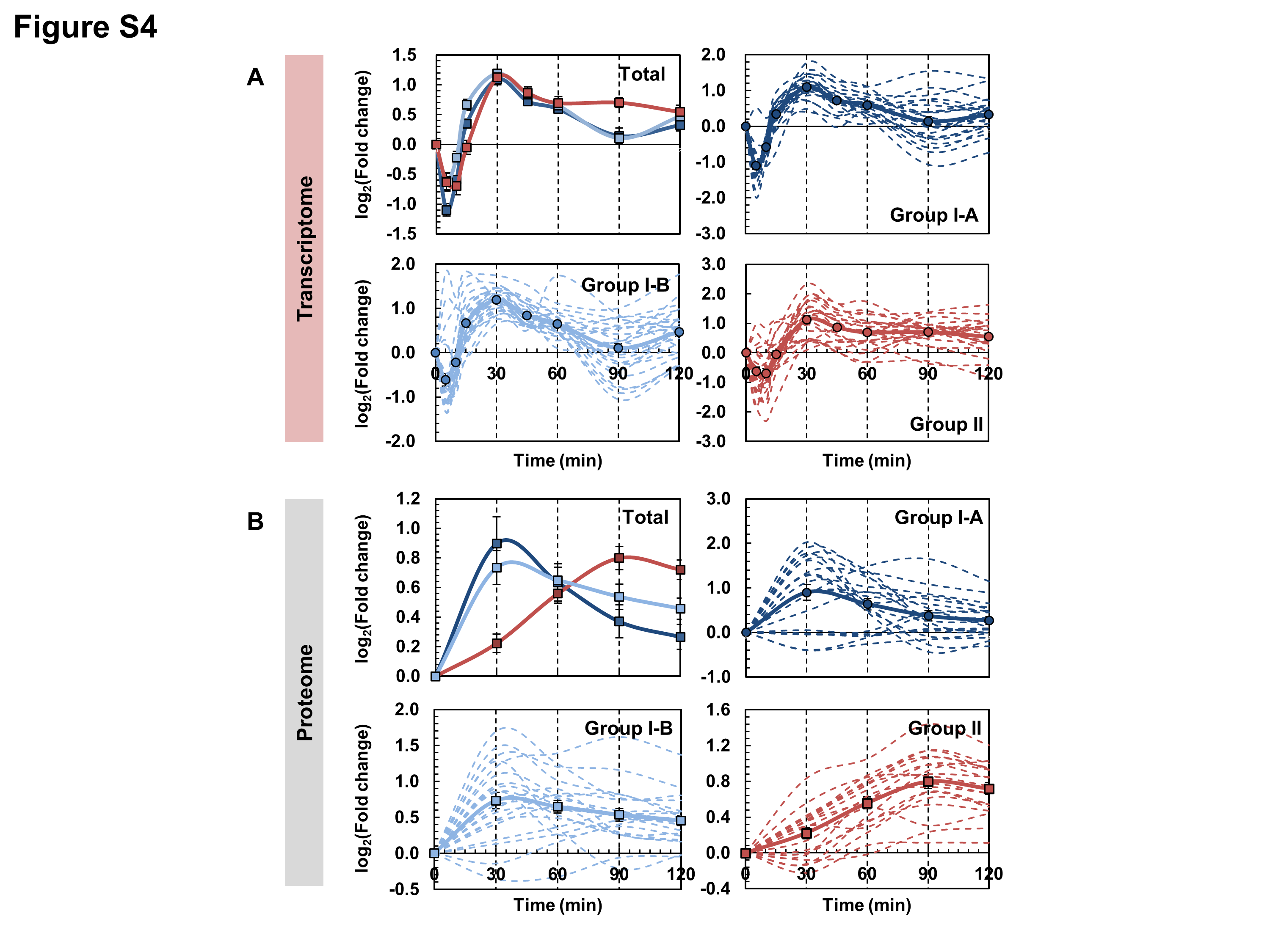

Supplement: Supplementary Figure 4 — Comparison of time-resolved mRNA and protein levels of cell motility-related genes. (A) Time-resolved mRNA levels of flagellar biosynthesis and chemotaxis-related genes for pathogenicity-activated Xoo cells. All genes in groups I-A, I-B, and II exhibited the lowest expression level at 5 min and the highest expression level at 30 min. (B) Time-resolved protein levels of flagellar biosynthesis and chemotaxis-related genes in pathogenicity-activated Xoo cells. Genes in groups I-A and I-B exhibited the highest expression level at 30 min, whereas those in group II exhibited the highest expression at 90 min. [file Image_4.TIF]

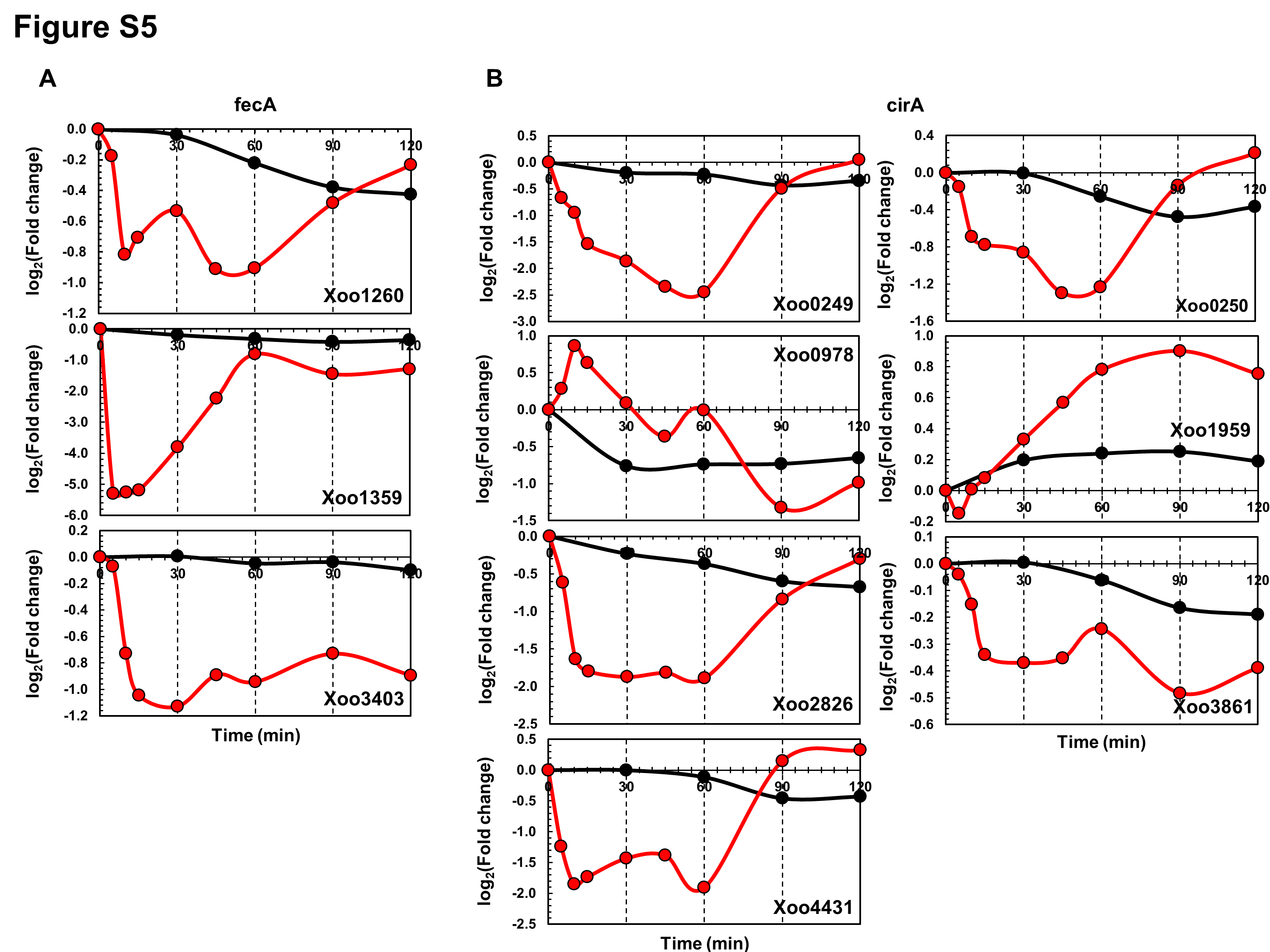

Supplement: Supplementary Figure 5 — Time-resolved mRNA and protein expression of iron uptake-related genes. Time-resolved mRNA (red) and protein (black) expression levels of iron uptake-related genes (A) FecA and (B) CirA. The Y-axis represents log2(fold change). [file Image_5.TIF]

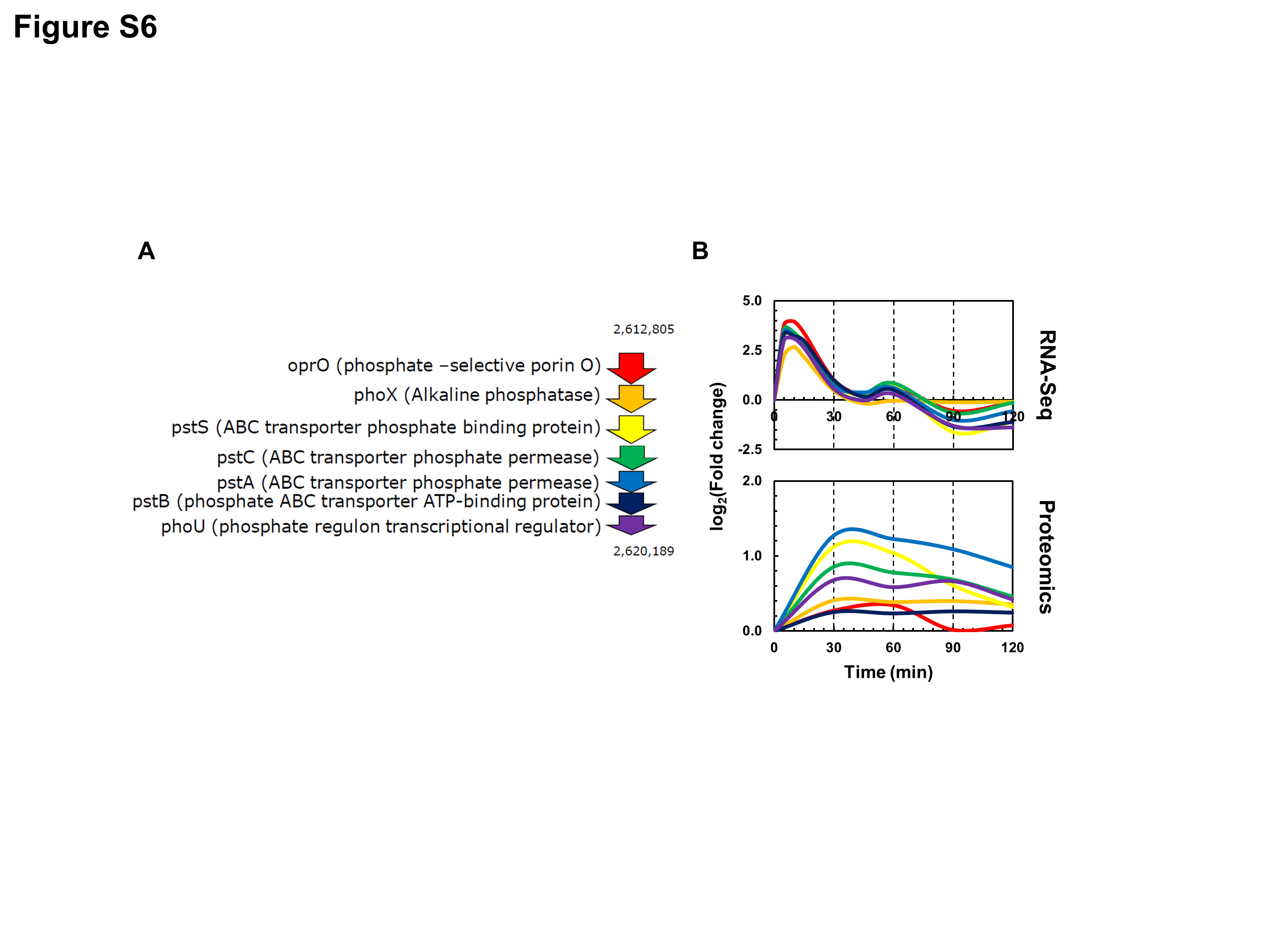

Supplement: Supplementary Figure 6 — Gene cluster and time-resolved mRNA and protein expression levels of phosphate uptake-related genes. (A) Gene cluster of the phosphate uptake regulation genes (OprO-PhoX-PstSCAB-PhoU). (B) Time-resolved mRNA and protein expression levels of phosphate uptake-related genes OprO, PhoX, PstSCAB, and PhoU. The line colors correspond to those of the arrows in (A). [file Image_6.TIF]

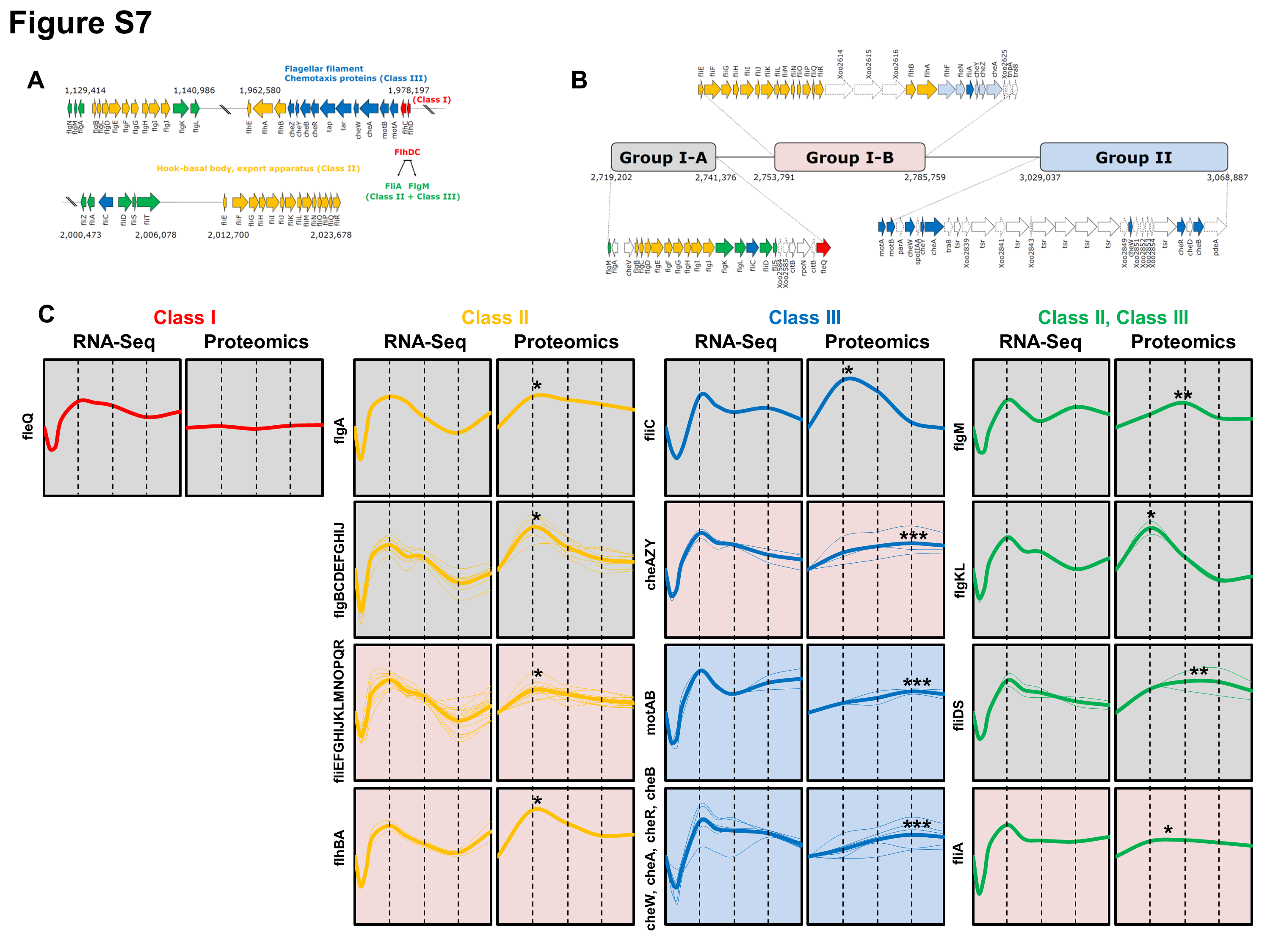

Supplement: Supplementary Figure 7 — Gene clusters of cell motility-related genes in E. coli and Xoo and time-resolved mRNA and protein expressions of Xoo genes. Gene cluster of cell motility-related genes of (A) E. coli and (B) Xoo. (C) Time-resolved mRNA and protein expression of cell motility-related genes in Xoo. The cell motility-related genes in E. coli are classified as class I (red), II (yellow), III (blue), and II + III (green). The ortholog genes in Xoo are indicated using the same color. For the proteome data, * indicates the expression peak at 30 min; **, between 30 and 90 min; ***, at 90 min. The Y-axis represents log2(fold change). [file Image_7.TIF]

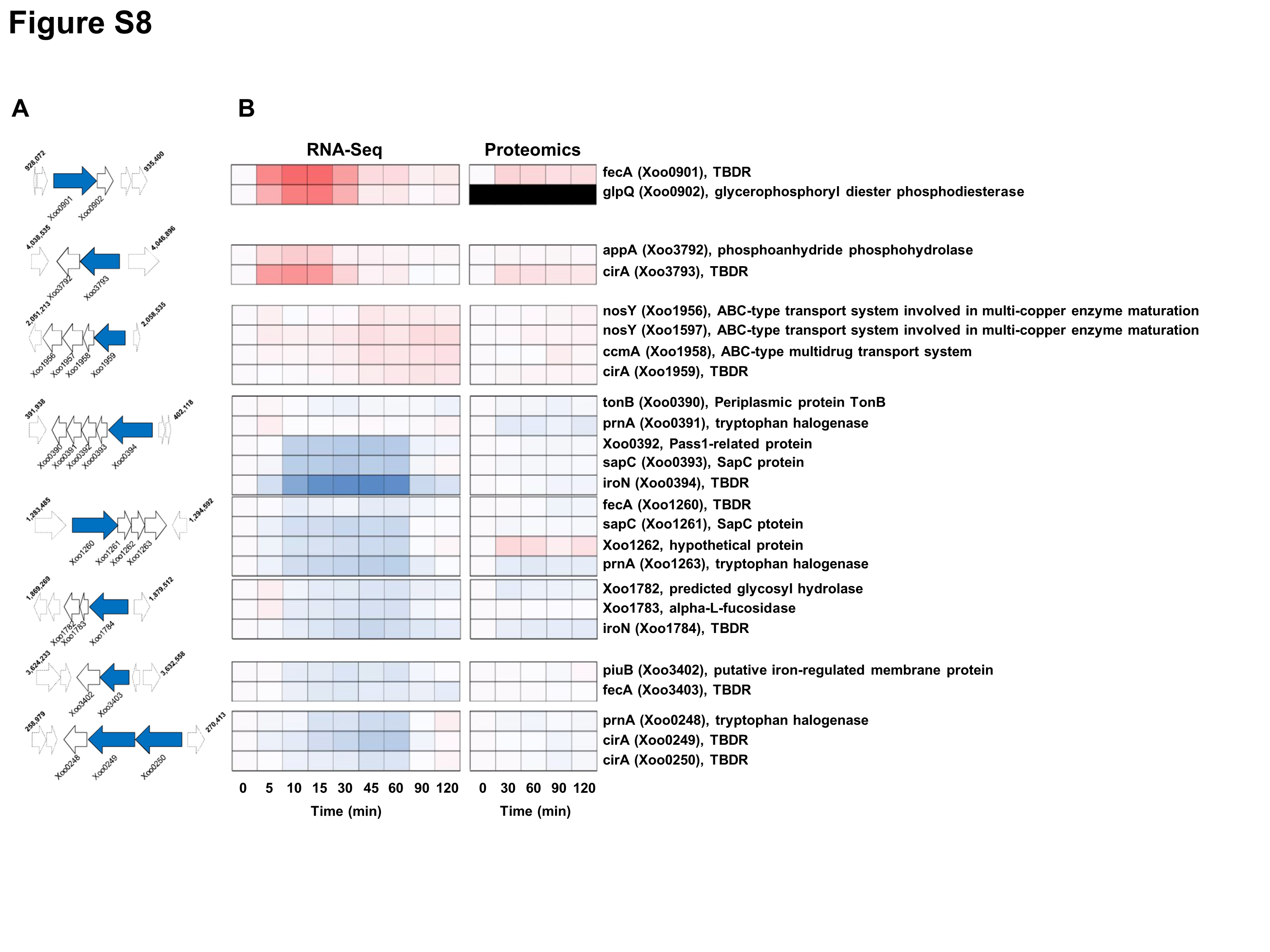

Supplement: Supplementary Figure 8 — Gene clusters and time-resolved mRNA and protein expressions of iron uptake-related genes in Xoo. (A) Gene cluster of iron uptake-related genes, labeled in blue. (B) Time-resolved mRNA and protein expression levels of iron uptake genes. Low to high expression is indicated by a change in color from blue to red. Black cells indicate undetected expression. [file Image_8.TIF]

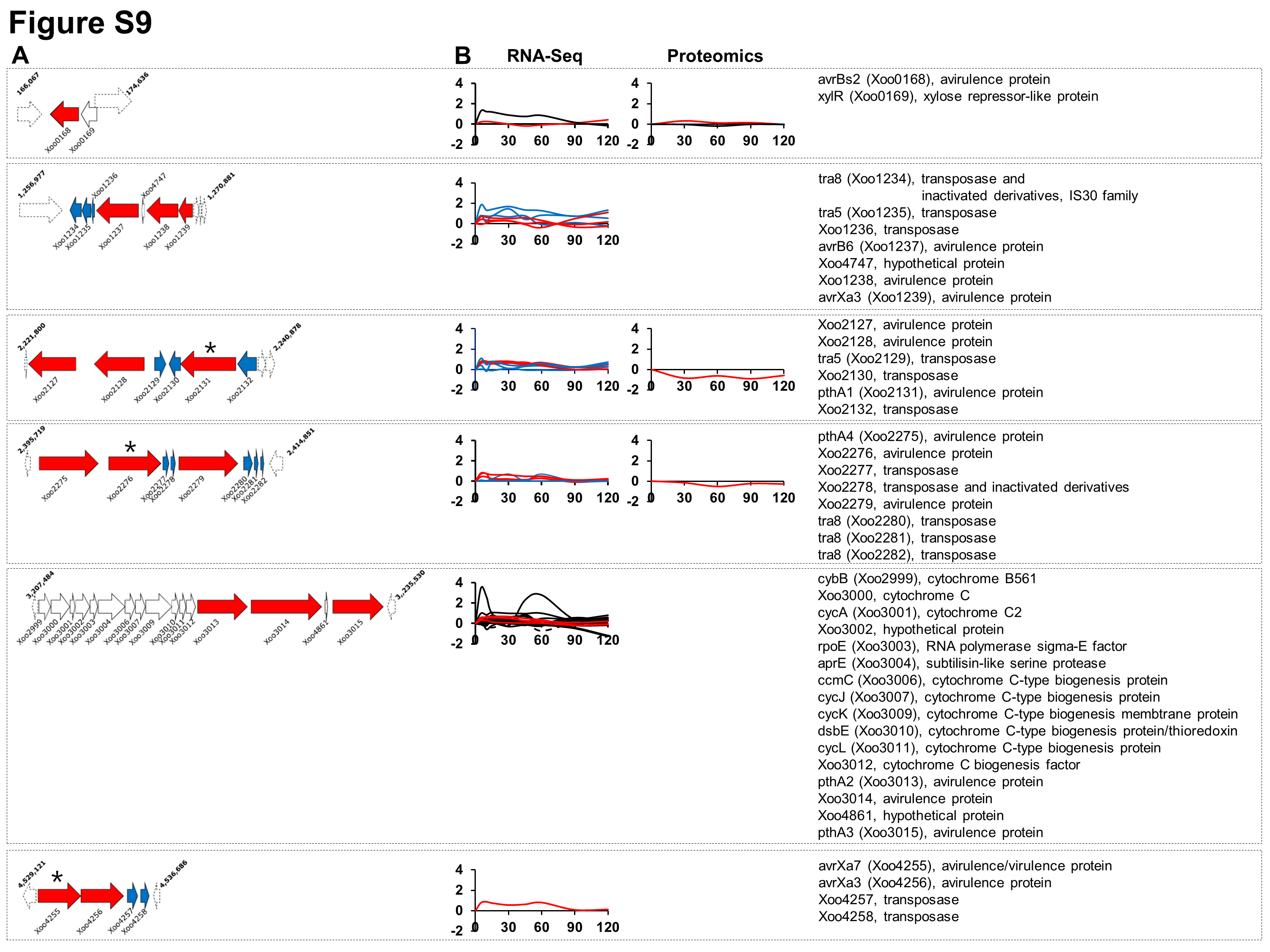

Supplement: Supplementary Figure 9 — Gene clusters and time-resolved mRNA and protein expressions of genes coding for effector molecules. (A) Gene cluster of effector genes, indicated in red. (B) Time-resolved mRNA and protein expressions of effector genes. Genes with available time-resolved proteomic data are labeled with *. Transposase genes are indicated in blue. [file Image_9.tif]

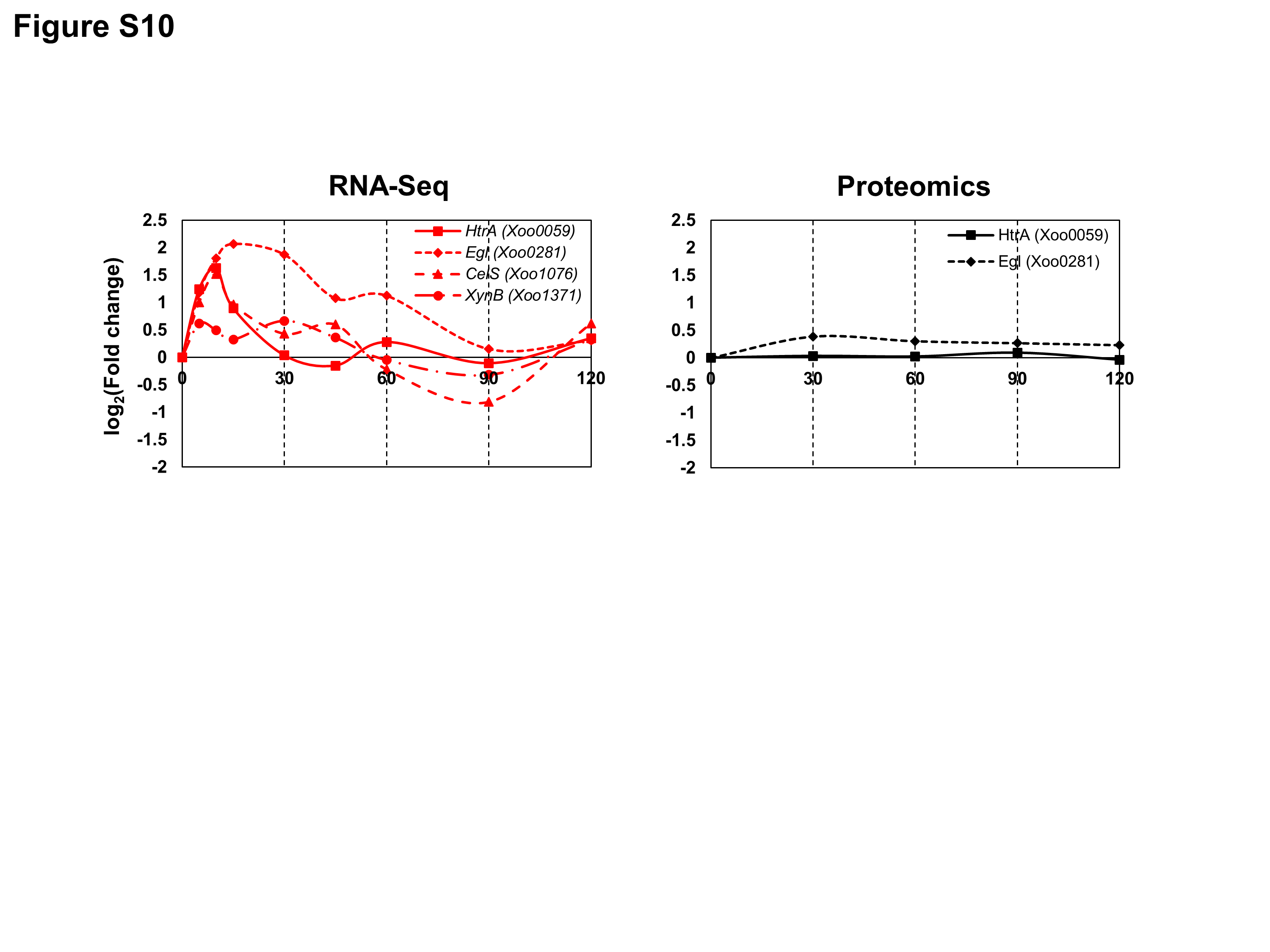

Supplement: Supplementary Figure 10 — Time-resolved mRNA and protein expression of plant cell wall degradation-related genes. Genes like HtrA (Xoo0059), Egl (Xoo0281), CelS (Xoo1076), and XynB (Xoo1371) were upregulated in transcriptome data. In proteome data, Egl (Xoo0281) was upregulated by 30% and HtrA (Xoo0059) was maintained at a similar level to that at 0 min. [file Image_10.TIF]

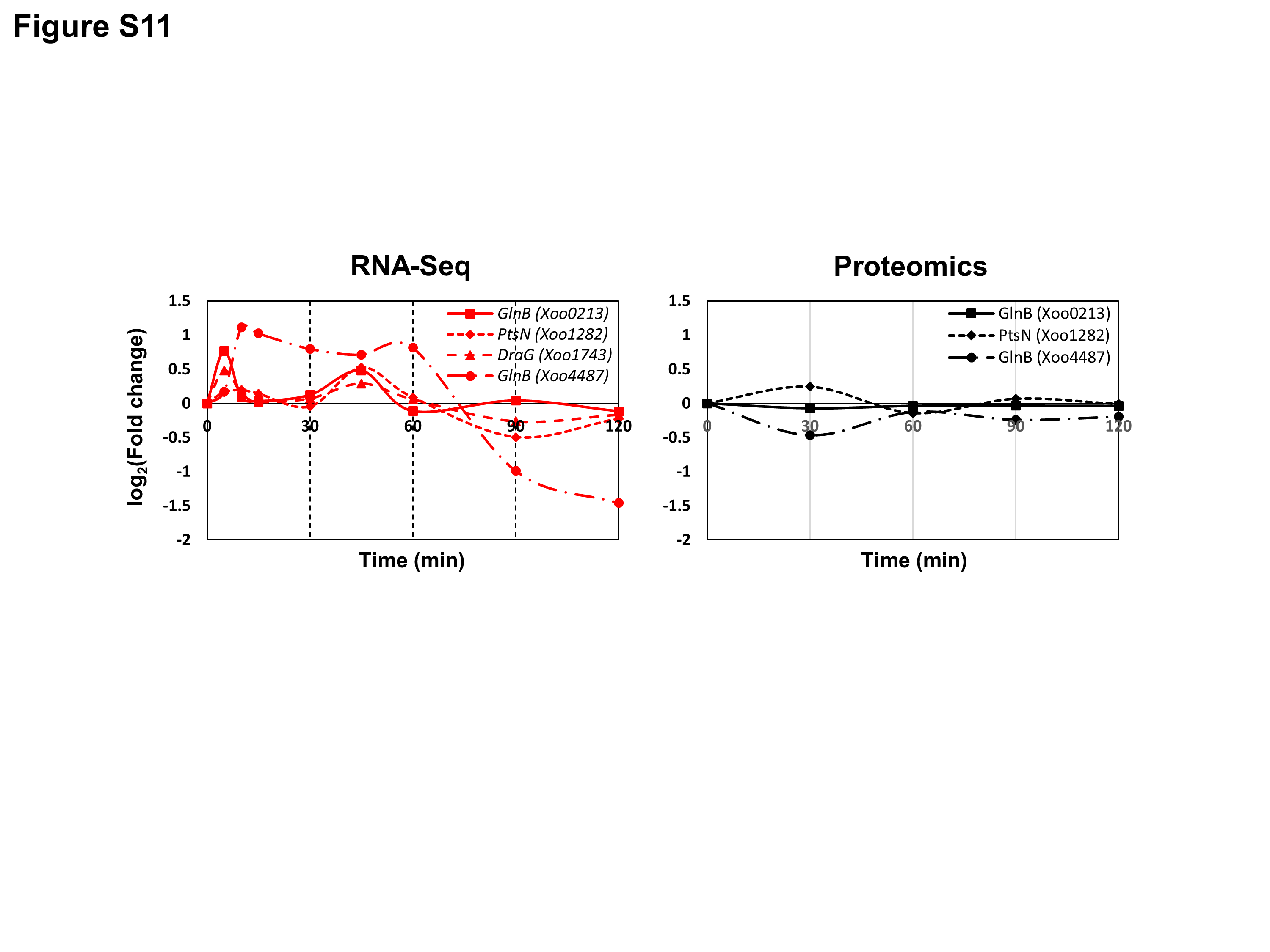

Supplement: Supplementary Figure 11 — Time-resolved mRNA and protein expression of nitrogen metabolism-related genes. Genes like GlnB (Xoo0213), GlnB (Xoo4487), PtsN (Xoo1282), and DraG (Xoo1743) were upregulated at 5–60 min in transcriptome data. In proteome data, PtsN (Xoo1282) was slightly upregulated at 30 min. [file Image_11.TIF]
